# Supplementary material for: Antimigratory Effect of Lipophilic Cations Derived from Gallic and Gentisic Acid and Synergistic Effect with 5-Fluorouracil on Metastatic Colorectal Cancer Cells: A New Synthesis Route
Source: Cancers (Basel). 2024 Aug 27;16(17):2980. doi: 10.3390/cancers16172980 (PMC11393949; doi:10.3390/cancers16172980)
Supplement: Supplementary file 1 [file cancers-16-02980-s001.zip › cancers-3148539-supplementary/cancers-3148539-supplementary/cancers-3148539-supplementary.pdf]

Supporting Information

SUPPORTING INFORMATION

Experimental Section

All solvents, including deuterated solvents, were purchased from Merck. Other reagents were from Aldrich, Merck or AK Scientific. Reactions run at room temperature were performed between 20 and 25 °C. Solvent evaporations were conducted under reduced pressure at temperatures less than 55 °C unless otherwise noted. Anhydrous Na_2SO_4 was used to dry organic layers after extraction, and it was removed by filtration through a cotton pad. The filtrate was concentrated and subjected to further purification protocols if necessary. All reactions were magnetically stirred and monitored for completion by normal phase Thin Layer Chromatography (TLC). Analytical TLC was performed on Merck precoated silica gel 60 F254 plates. TLC was visualized by one of the following methods: use of UV light (254 nm) or immersion into an iodine vapor or vanillin stain solution followed by heating. Column chromatography was carried out under positive pressure using 40-63 µm silica gel (Merck) and the indicated solvents [v:v; used without purification]. NMR spectra were recorded on a Bruker Avance 400 MHz spectrometer. All chemical shifts in NMR experiments are reported as ppm downfield from TMS. The following calibrations were used: $CDCl_3$ δ = 7.26 and 77.0 ppm for ^1H NMR and ^{13}C NMR respectively and DMSO-d_6 δ = 2.50 and 39.52 ppm for ^1H NMR and ^{13}C NMR respectively. Coupling constants (J) are given in Hertz (Hz), and the following abbreviations are used to describe the signal multiplicity: s (singlet), bs (broad singlet), d (doublet), dd (doublet of doublet), t (triplet), td (triplet of doublets), q (quartet), quint (quintet) and m (multiplet). All NMR spectra were processed and analyzed using MestRe Nova 14.1.2. Melting points were determined on a Reichert Galen III hot plate microscope apparatus and are uncorrected. High resolution mass spectrometric (HRMS) were performed using a Bruker "Compact" quadrupole time-of-flight mass spectrometry (qTOF-MS, Germany) coupled with an Apollo II ion funnel electrospray ionization (ESI) source from the "Unidad de Secuenciación y Tecnologías Ómicas" (Facultad de Ciencias Biológicas, Pontificia Universidad Católica de Chile), Chile.

Abbreviations

Aq.= aqueous solution

BnCl = benzyl chloride

Calcd. = calculated

DCC = *N*,*N'*-dicyclohexylcarbodiimide

DMAP = 4-dimethylaminopyridine

DMF = *N*,*N*-dimethylformamide

DMSO = dimethyl sulfoxide

DCM = dichloromethane

EtOAc = ethyl acetate

EtOH = ethanol

Eq = equivalent

HRMS = High Resolution Mass Spectrometry

MeOH = methanol

NMR = Nuclear Magnetic Resonance

PPh_3 = triphenylphosphine

Sat.= saturated

THF = tetrahydrofuran

Construction of a unit fully Galic Acid (GA) protected in the hydroxyl groups (Scheme S1).

Scheme S1 illustrate the detailed synthetic route of the protected compound **1** according to the procedure described in the previous study by Ren and co-worked.[1] The gallic acid (**GA**) was selected as the starting material, which was subject to Fischer esterification with MeOH to give methyl gallate (**GA-S1**) in a 98% yield. With the key ester intermediate **GA-S1** in hand, the Ar-OH groups are protected with benzyl chloride (BnCl), K_2CO_3 and catalytic amounts of KI in acetone at reflux overnight, that the expected product in 97% yield. Finally, the compound **GA-S2** was treated with NaOH in ethanol by refluxing and acid **1** was obtained in 85% yield (81% overall yield in 3 steps).

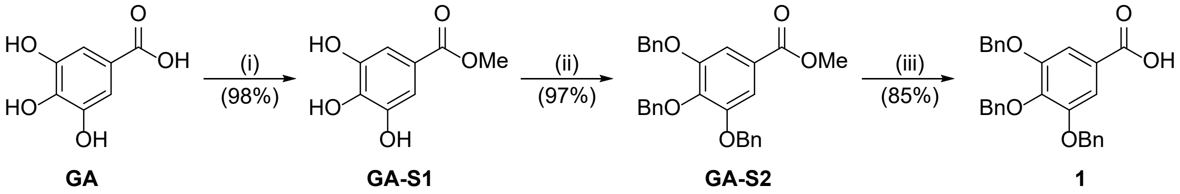

Scheme 1[a]

[a] Reagents and conditions: (i) Conc. H_2SO_4, MeOH, reflux, 12 h; (ii) BnCl, K_2CO_3, KI, acetone, reflux, overnight; (iii) NaOH, EtOH, reflux, 3 h.

methyl 3,4,5-trihydroxybenzoate (methyl gallate; GA-S1)

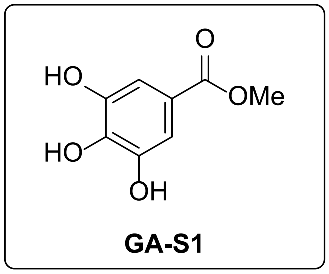

To a stirred solution of **GA** (5.00 g; 28.29 mmol) in MeOH (100 mL), concentrated H_2SO_4 (2.50 mL) was added in one portion at room temperature. The resulting solution was stirred at reflux for 12 hours. After this time, the reaction was concentrated under reduced pressure. The crude product was resuspended in 150 mL of sat. aq. $NaHCO_3$, and the aqueous layer was extracted twice with EtOAc. The combined extracts were washed with water (50 mL) and brine (30 mL), dried over Na_2SO_4, filtered, and concentrated to afford a **GA-S1** (4.90 g, 98% yield) as a pale

white solid. ^{1}H NMR (400 MHz, DMSO-d_6) δ 9.20 (brs, 3H), 6.94 (s, 2H), 3.74 (s, 3H). Spectroscopic data are in agreement with literature values.[2]

methyl 3,4,5-tris(benzyloxy)benzoate (GA-S2)

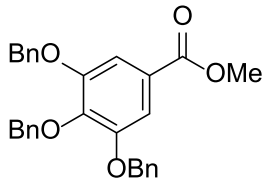

GA-S2

A mixture of **GA-S1** (1.47 g; 8.00 mmol; 1 eq), finely granulated K_2CO_3 (6.63 g; 48 mmol; 6 eq) and KI (1.99 g; 12 mmol; 1.5 eq) in acetone (40 mL) was stirred at room temperature for 20 min. Then, benzyl chloride (3.04 mL; 26.40 mmol; 3.3 eq) was added in one portion at room temperature. The resulting suspension was stirred at reflux for overnight. After this time, the reaction was cooled to room temperature and the resulting mixture filtrated with pad of celite, washed with DCM and concentrated under reduced pressure. The crude product was subjected to silica gel column chromatography (n-hexane/EtOAc, 1:3 as the eluent) to provide **GA-S2** (3.51g, 97% yield) as a white solid. ^{1}H NMR (400 MHz, DMSO-d_6) δ 7.52 – 7.22 (m, 17H), 5.18 (s, 4H), 5.05 (s, 2H), 3.83 (s, 3H). Spectroscopic data are in agreement with literature values.[3]

3,4,5-tris(benzyloxy)benzoic acid (1)

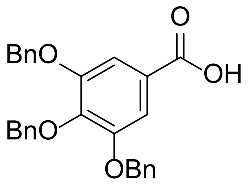

1

To a stirred solution of **GA-S2** (5.67 g; 12.47 mmol) in EtOH (112 mL), finely pulverized NaOH (748 mg; 18.71 mmol; 1.5 eq) was added in one portion at room temperature. The resulting mixture was stirred at reflux for 3 hours. After this time, the reaction was cooled to room temperature, water (100 mL) was added and acidified with concentrated aq. HCl (pH= 2). The precipitate formed is vacuum filtered to give a pale white solid that was recrystallized from MeOH/ EtOAc (4:1, v/v) to yield **1** (4.69 g, 85%) as white needles. ^{1}H NMR (400 MHz, DMSO-d_6) δ 12.93 (s, 1H), 7.53 – 7.22 (m, 17H), 5.18 (s, 4H), 5.04 (s, 2H). Spectroscopic data are in agreement with literature values.[4]

Construction of a unit fully Gentisic Acid (GE) protected in the hydroxyl groups (Scheme S2).

Scheme S2 illustrate the detailed synthetic route of the protected compound **2** adapting and slightly modifying the procedure described by Ren and co-worked (previously used in the protection of **GA**).[1] The acid **2** protected on aromatic hydroxyls was obtained in 83% overall yield in 3 steps.

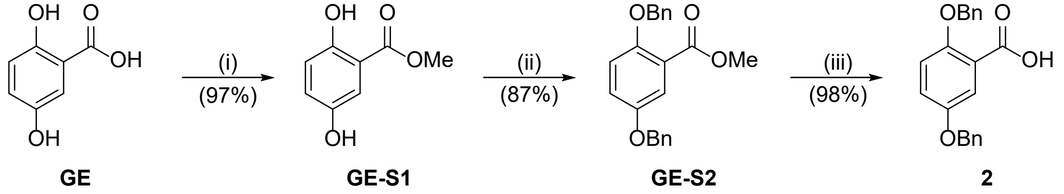

Scheme 2[a]

[a] Reagents and conditions: (i) Conc. H_2SO_4, MeOH, reflux, 12 h; (ii) BnCl, K_2CO_3, KI, acetone, reflux, overnight; (iii) NaOH, EtOH, reflux, 3 h.

methyl 2,5-dihydroxybenzoate (GE-S1)

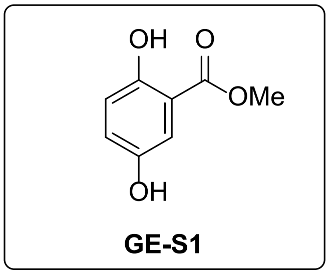

To a stirred solution of **GE** (5.00 g; 32.44 mmol) in MeOH (150 mL), concentrated H_2SO_4 (2.50 mL) was added in one portion at room temperature. The resulting solution was stirred at reflux for 12 hours. After this time, the reaction was concentrated under reduced pressure. The crude product was resuspended in 150 mL of sat. aq. $NaHCO_3$, and the aqueous layer was extracted twice with EtOAc. The combined extracts were washed with water (50 mL) and brine (30 mL), dried over Na_2SO_4, filtered, and concentrated to afford an **GE-S1** (5.27 g, 97% yield) as a pale white solid. ^{1}H NMR (400 MHz, DMSO-d_6) δ 9.92 (s, 1H), 9.20 (s, 1H), 7.15 (d, J = 3.0 Hz, 1H), 6.98 (dd, J = 8.9, 3.1 Hz, 1H), 6.82 (d, J = 8.9 Hz, 1H), 3.87 (s, 3H). Spectroscopic data are in agreement with literature values.[5]

methyl 2,5-bis(benzyloxy)benzoate (GE-S2)

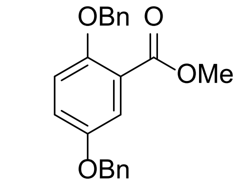

GE-S2

A mixture of **GA-S1** (1.35 g; 8.00 mmol; 1 eq), finely granulated K_2CO_3 (4.42 g; 32 mmol; 4 eq) and KI (1.33 g; 8 mmol; 1 eq) in acetone (50 mL) was stirred at room temperature for 20 min. Then, benzyl chloride (2.03 mL; 17.60 mmol; 2.2 eq) was added in one portion at room temperature. The resulting suspension was stirred at reflux for overnight. After this time, the reaction was cooled to room temperature and the resulting mixture filtrated with pad of celite, washed with DCM and concentrated under reduced pressure. The crude product was subjected to silica gel column chromatography (n-hexane/ EtOAc, 1:4 as the eluent) to provide **GE-S2** (2.43g, 87% yield) as a white solid. ^{1}H NMR (400 MHz, DMSO-d_6) δ 7.51 – 7.13 (m, 13H), 5.13 (s, 2H), 5.08 (s, 2H), 3.80 (s, 3H). Spectroscopic data are in agreement with literature values.[6]

2,5-bis(benzyloxy)benzoic acid (2)

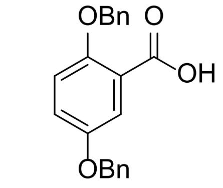

2

To a stirred solution of **GA-S2** (5.67 g; 12.47 mmol) in EtOH (112 mL), finely pulverized NaOH (748 mg; 18.71 mmol; 1.5 eq) was added in one portion at room temperature. The resulting mixture was stirred at reflux for 3 hours. After this time, the reaction was cooled to room temperature, water (100 mL) was added and acidified with concentrated aq. HCl (pH= 2). The precipitate formed is vacuum filtered, washed with water and to give **2** (4.69 g, 85%) as white needles. ^{1}H NMR (400 MHz, DMSO-d_6) δ 12.73 (s, 1H), 7.76 – 7.00 (m, 13H), 5.12 (s, 2H), 5.08 (s, 2H). Spectroscopic data are in agreement with literature values.[7]

Synthesis of 10-bromodecan-1-ol (S1)

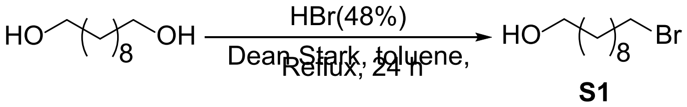

This procedure was adapted from the work of Kadam and Sudhakar.[8] To a stirred solution of decane-1,10-diol (2.50 g; 14.34 mmol) in toluene (150 mL), the aqueous solution HBr (48%; 1.79 mL; 15.77 mmol; 1.1 eq) was added in one portion at room temperature. Then, the reaction was refluxed equipped with a Dean-Stark apparatus for 24 h. The resulting solution was washed whit sat. aq. $NaHCO_3$, water and brine, dried over Na_2SO_4, filtered, and concentrated under reduced pressure. The crude product was subjected to silica gel column chromatography (n-hexane/ EtOAc, 1:4 as the eluent) to provide **S1** (2.81 g, 83%) as a clear. ^{1}H NMR (400 MHz, $CDCl_3$) δ 3.62 (td, J = 6.7, 1.8 Hz, 2H), 3.39 (td, J = 6.8, 0.8 Hz, 2H), 1.84 (quint, J = 7.0 Hz, 2H), 1.55 (quint, J = 7.4, 7.0 Hz, 2H), 1.45 – 1.23 (m, 12H). Spectroscopic data matched that previously reported.[9]

General methodology of Steglich esterification for the formation of intermediates 3 and 4 (Scheme S3).

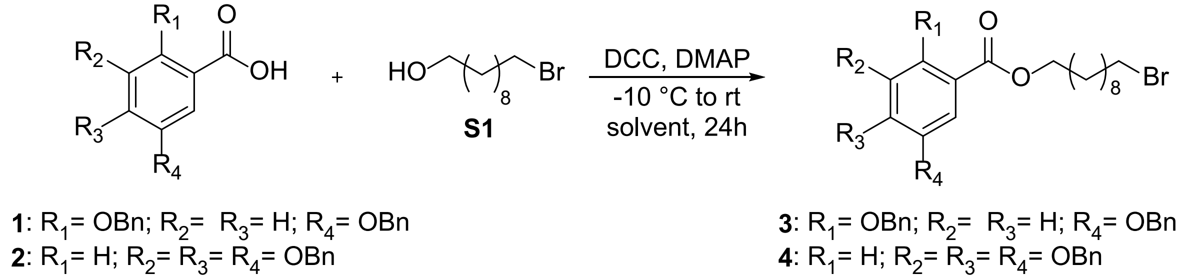

Scheme S3

General Procedure: A mixture of carboxylic acid protected in the hydroxyl groups (**1** or **2**; 3.35 mmol; 1 eq), **S1** (795 mg; 3.35 mmol; 1 eq) and DMAP (22 mg; 179 µmol; 0.05 eq) in in the appropriate solvent (30 mL; **1** in DCM and **2** in DMF) was stirred at -10 °C (cooling bath: acetone/ice/NaCl) for 15 min. Thereafter, DCC (691 mg; 3.35 mmol; 1 eq) was added

in one portion at same temperature and the reaction was stirred at -10 °C for 30 min. After this time, the reaction was warmed very slowly to room temperature and stirred for 24 h. Finally, the precipitate formed was discarded by filtration and the crude product was concentrated under reduced pressure. The crude product was subjected to silica gel column chromatography (n-hexane/ EtOAc, 1:3 as the eluent) to afford ester **3** or **4**.

10-bromodecyl 2,5-bis(benzyloxy)benzoate (3)

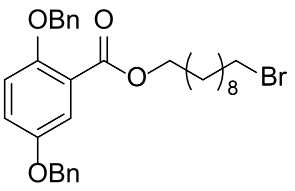

3

Clear oil (1.65 g, 89%). ^{1}H NMR (400 MHz, DMSO-d_6) δ 7.52 – 7.11 (m, 13H), 5.11 (s, 2H), 5.07 (s, 2H), 4.19 (t, J = 6.5 Hz, 2H), 3.48 (t, J = 6.7 Hz, 2H), 1.75 (quint, J = 6.8 Hz, 2H), 1.61 (quint, J = 6.6 Hz, 2H), 1.37 – 1.17 (m, 12H). ^{13}C NMR (101 MHz, DMSO-d_6) δ 165.6, 151.8, 151.3, 137.1, 136.9, 128.4, 128.2, 127.8, 127.6, 127.1, 121.6, 119.6, 116.4, 115.8, 70.4, 69.8, 64.6, 35.1, 32.2, 28.8, 28.6, 28.1, 28.0, 27.5, 25.4. HRMS for $C_{31}H_{38}BrO_4$ $[M+H]^+$: Found: 553.1937; Calcd.: 553.1948.

10-bromodecyl 3,4,5-tris(benzyloxy)benzoate (4)

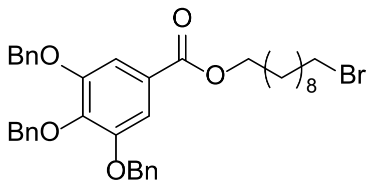

4

White solid (928 mg, 42%). ^{1}H NMR (400 MHz, DMSO-d_6) δ 7.49 – 7.24 (m, 17H), 5.17 (s, 4H), 5.05 (s, 2H), 4.22 (t, J = 6.3 Hz, 2H), 3.48 (t, J = 6.7 Hz, 2H), 1.80 – 1.63 (m, 4H), 1.38 – 1.22 (m, 12H). ^{13}C NMR (101 MHz, DMSO-d_6) δ 165.2, 152.1, 141.3, 137.3, 136.7, 128.4, 128.2, 128.1, 127.9, 127.6, 125.0, 108.1, 74.2, 70.3, 64.8, 35.1, 32.2, 28.8, 28.6, 28.1, 28.1, 27.5, 25.4. HRMS for $C_{38}H_{44}BrO_5$ $[M+H]^+$: Found: 659.2380; Calcd.: 659.2367.

General methodology of *O*-debenzylation in the hydroxyl groups for the formation of intermediates 5 and 6 (Scheme S4).

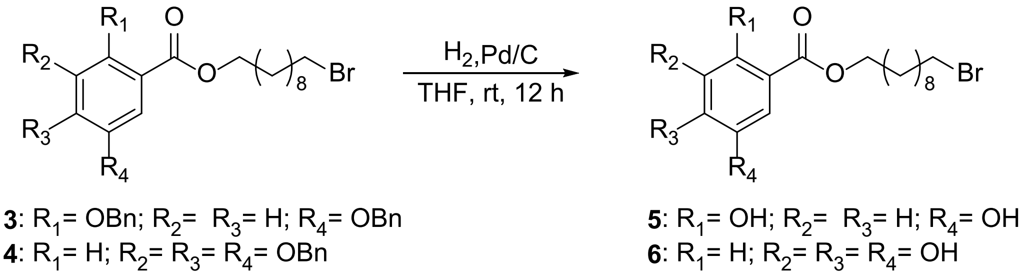

Scheme S4

General Procedure: To a solution of *O*-protected ester (**3** or **4**; 1.50 mmol; 1 eq) in THF (15 mL) were added 10% palladium on carbon catalyst (30% in weight is added). Then, the flask was put under a H_2 atmosphere with three cycles of vacuum/H_2 and the mixture was stirred at room temperature under atmospheric pressure of H_2 for 12 h. After this time, the resulting mixture filtrated with pad of celite, washed with THF and concentrated under reduced pressure to afford unprotected ester **5** or **6** in quantitative conversion. To store for the sequent reaction, in the inert atmosphere. No further treatments were applied prior to its using.

10-bromodecyl 2,5-dihydroxybenzoate (5)

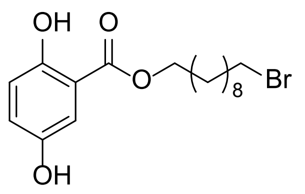

5

Colorless viscous oil (559 mg, quantitative yield). ^{1}H NMR (400 MHz, DMSO-d_6) δ 10.0 (s, 1H), 9.2 (s, 1H), 7.2 (d, J = 3.0 Hz, 1H), 7.0 (dd, J = 9.0, 3.1 Hz, 1H), 6.8 (d, J = 8.9 Hz, 1H), 4.3 (t, J = 6.5 Hz, 2H), 3.5 (t, J = 6.7 Hz, 2H), 1.9 – 1.6 (m, 4H), 1.5 – 1.2 (m, 12H). ^{13}C NMR (101 MHz, DMSO-d_6) δ 169.0, 153.4, 149.6, 123.9, 118.1, 114.0, 112.4, 65.1, 35.1, 32.2, 28.8, 28.8, 28.6, 28.1, 28.0, 27.5, 25.4. HRMS for $C_{17}H_{26}BrO_4$ $[M + H]^+$: Found: 373.1007; Calcd.: 373.1009.

10-bromodecyl 3,4,5-trihydroxybenzoate (6)

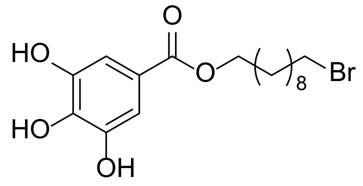

6

Colorless viscous oil (583 mg, quantitative yield). ^{1}H NMR (400 MHz, DMSO-d_6) δ 9.2 (brs, 2H), 8.9 (brs, 1H), 6.9 (s, 2H), 4.1 (t, J = 6.5 Hz, 2H), 3.5 (t, J = 6.7 Hz, 2H), 1.8 (quint, J = 6.8 Hz, 2H), 1.6 (quint, J = 6.6 Hz, 2H), 1.4 – 1.2 (m, 12H). ^{13}C NMR (101 MHz, DMSO-d_6) δ 165.9, 145.5, 138.3, 119.6, 108.5, 63.9, 35.1, 32.3, 28.9, 28.8, 28.7, 28.3, 28.1, 27.5, 25.5. HRMS for $C_{17}H_{26}BrO_5$ $[M + H]^+$: Found: 389.0961; Calcd.: 389.0958.

General methodology for the formation of delocalized lipophilic cations (GE-C10-TPP$^+$ and GA-C10-TPP$^+$) *via* SN2 nucleophilic substitution (Scheme S5).

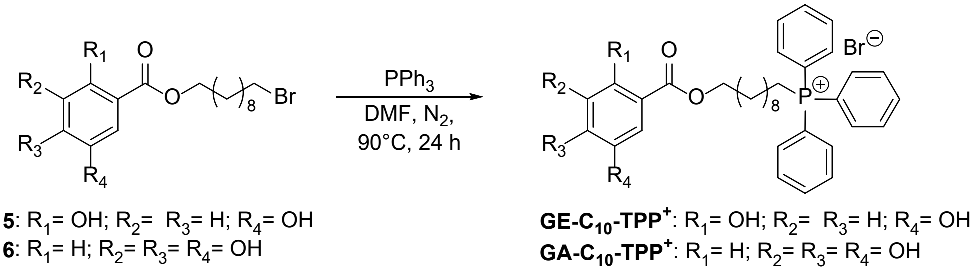

Scheme S5

General Procedure: To a stirred solution of unprotected ester (**5** or **6**; 1.40 mmol; 1 eq) in DMF (4 mL) were added PPh_3 (734 mg; 2.80 mmol; 2 eq) at room temperature under N_2. The resulting mixture was stirred at 90 °C for 14 h under N_2 atmosphere. Then, the crude product was concentrated under reduced pressure. The crude product was subjected to silica gel column chromatography (MeOH/ EtOAc, 1:4 as the eluent) to afford the delocalized lipophilic cation (**GE-C10-TPP$^+$** or **GA-C10-TPP$^+$**).

(10-((2,5-dihydroxybenzoyl)oxy)decyl)triphenylphosphonium bromide (GE-C10-TPP⁺)

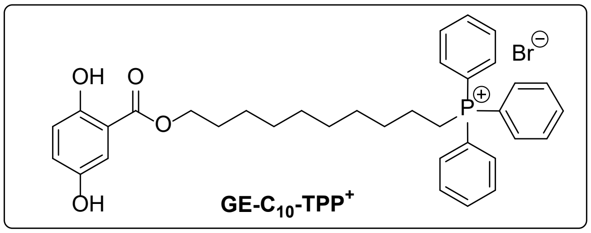

Colourless oil (810 mg, 91%). ^{1}H NMR (400 MHz, DMSO-d_6) δ 10.0 (s, 1H), 9.2 (brs, 1H), 7.9 – 7.7 (m, 15H), 7.2 (d, J = 3.0 Hz, 1H), 7.0 (dd, J = 8.9, 3.0 Hz, 1H), 6.8 (d, J = 8.9 Hz, 1H), 4.3 (t, J = 6.5 Hz, 2H), 1.7 (quint, J = 6.6 Hz, 2H), 1.6 – 1.1 (m, 16H). ^{13}C NMR (101 MHz, DMSO-d_6) δ 168.9, 153.3, 149.5, 134.8 (d, J = 2.9 Hz), 133.6 (d, J = 10.0 Hz), 130.2 (d, J = 12.4 Hz), 123.9, 119.0, 118.1 (d, J = 6.1 Hz), 114.0, 112.4, 65.0, 29.7 (d, J = 16.5 Hz), 28.7, 28.5 (d, J = 7.6 Hz), 28.0, 27.9, 25.3, 21.7 (d, J = 4.2 Hz), 20.4, 19.9. Spectroscopic data matched that previously reported.[10]

triphenyl(10-((3,4,5-trihydroxybenzoyl)oxy)decyl)phosphonium bromide (GA-C10-TPP⁺)

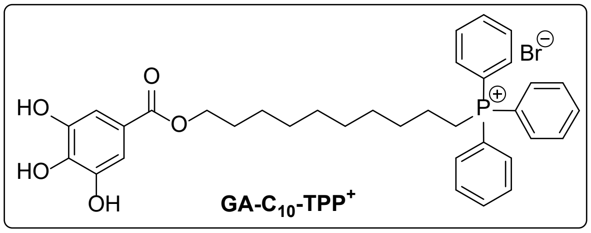

Colourless oil (564 mg, 62%). ^{1}H NMR (400 MHz, DMSO-d_6) δ 7.9 – 7.7 (m, 15H), 7.0 (s, 2H), 4.1 (t, J = 6.5 Hz, 2H), 1.6 (quint, J = 6.7 Hz, 2H), 1.6 – 1.1 (m, 16H). ^{13}C NMR (101 MHz, DMSO-d_6) δ 165.9, 145.5, 138.4, 134.8 (d, J = 2.9 Hz), 133.6 (d, J = 10.0 Hz), 130.2 (d, J = 12.4 Hz), 119.5, 119.0, 118.1, 108.5, 63.9, 29.7 (d, J = 16.1 Hz), 28.7, 28.6 (d, J = 2.6 Hz), 28.2, 28.0, 25.4, 21.7 (d, J = 4.1 Hz), 20.4, 19.9. Spectroscopic data matched that previously reported.[10]

References

(1) Ren, Y.; Himmeldirk, K.; Chen, X. Synthesis and Structure−Activity Relationship Study of Antidiabetic Penta-O-Galloyl-d-Glucopyranose and Its Analogues. *J. Med. Chem.* **2006**, *49* (9), 2829–2837. https://doi.org/10.1021/jm060087k.

(2) Zou, J.; Tao, F.; Jiang, M. Optical Switching of Self-Assembly and Disassembly of Noncovalently Connected Amphiphiles. *Langmuir* **2007**, *23* (26), 12791–12794. https://doi.org/10.1021/la702815h.

(3) Rajamalli, P.; Prasad, E. Low Molecular Weight Fluorescent Organogel for Fluoride Ion Detection. *Org. Lett.* **2011**, *13* (14), 3714–3717. https://doi.org/10.1021/ol201325j.

(4) Dhingra, M. S.; Dhingra, S.; Chadha, R.; Singh, T.; Karan, M. Design, Synthesis, Physicochemical, and Pharmacological Evaluation of Gallic Acid Esters as Non-Ulcerogenic and Gastroprotective Anti-Inflammatory Agents. *Med. Chem. Res.* **2014**, *23* (11), 4771–4788. https://doi.org/10.1007/s00044-014-1041-x.

(5) Borges, R. S.; Castle, S. L. The Antioxidant Properties of Salicylate Derivatives: A Possible New Mechanism of Anti-Inflammatory Activity. *Bioorg. Med. Chem. Lett.* **2015**, *25* (21), 4808–4811. https://doi.org/10.1016/j.bmcl.2015.07.001.

(6) Mondal, M.; Puranik, V. G.; Argade, N. P. Facile Synthesis of 1,3,7-Trihydroxyxanthone and Its Regioselective Coupling Reactions with Prenal: Simple and Efficient Access to Osajaxanthone and Nigrolineaxanthone F. *J. Org. Chem.* **2006**, *71* (13), 4992–4995. https://doi.org/10.1021/jo0606655.

(7) Kawa, M.; Takahagi, T. Improved Antenna Effect of Terbium(III)-Cored Dendrimer Complex and Green-Luminescent Hydrogel by Radical Copolymerization. *Chem. Mater.* **2004**, *16* (11), 2282–2286. https://doi.org/10.1021/cm034873e.

(8) Kadam, V. D.; Sudhakar, G. Total Synthesis of Motualevic Acids A–F, (E) and (Z)-Antazirines. *Tetrahedron* **2015**, *71* (7), 1058–1067. https://doi.org/10.1016/j.tet.2014.12.092.

(9) de Oliveira, A. J.; Souza, I. T.; Bernardo, V. B.; Santos, L. C.; de Lima, M. R. F.; Goulart, H. F.; Goulart Santana, A. E. Monobromination of α,ω-Diols: Highly Efficient Preparation of Synthetic Intermediates. *ChemistrySelect* **2019**, *4* (36),

10843–10845. https://doi.org/10.1002/slct.201901879.

(10) Catalán, M.; Castro-Castillo, V.; Gajardo-de la Fuente, J.; Aguilera, J.; Ferreira, J.; Ramires-Fernandez, R.; Olmedo, I.; Molina-Berríos, A.; Palominos, C.; Valencia, M.; Domínguez, M.; Souto, J. A.; Jara, J. A. Continuous Flow Synthesis of Lipophilic Cations Derived from Benzoic Acid as New Cytotoxic Chemical Entities in Human Head and Neck Carcinoma Cell Lines. *RSC Med. Chem.* **2020**, *11* (10), 1210–1225. https://doi.org/10.1039/D0MD00153H.

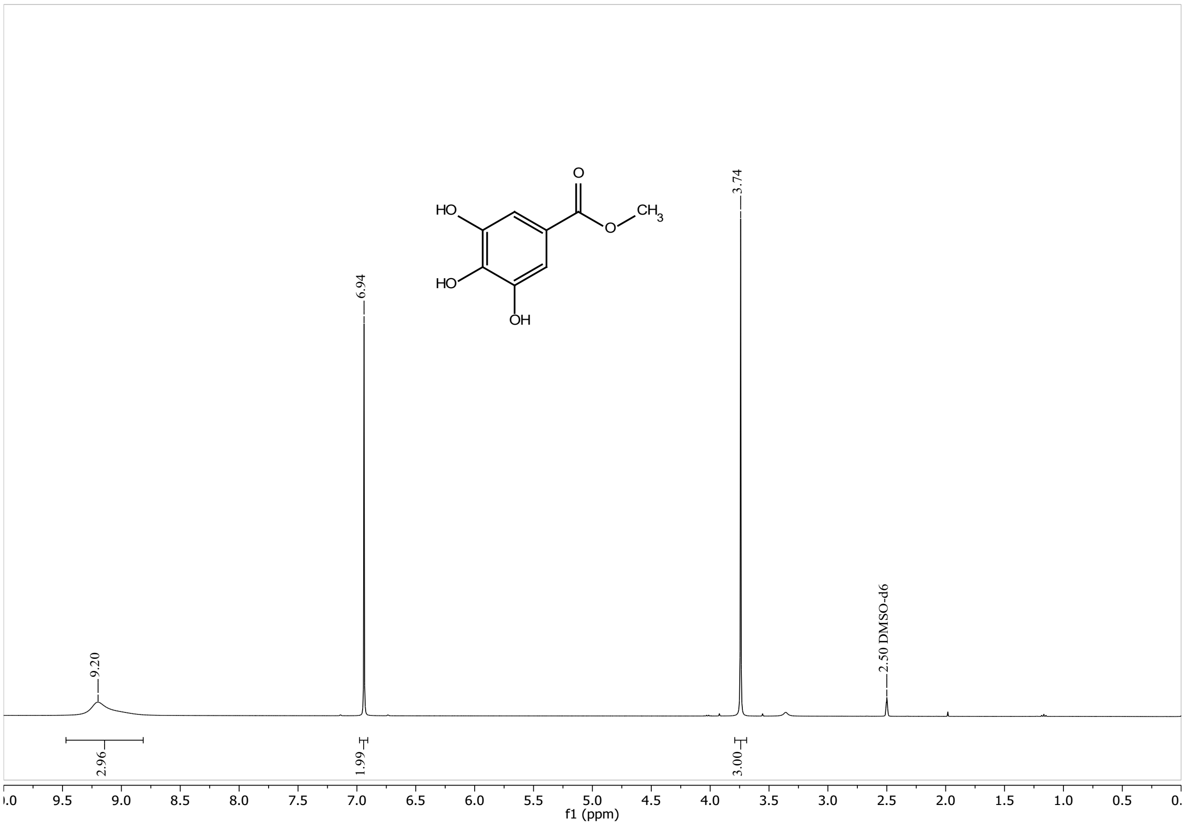

Figure S1. ^{1}H-NMR spectrum of **GA-S1** (DMSO-d_6, 400 MHz)

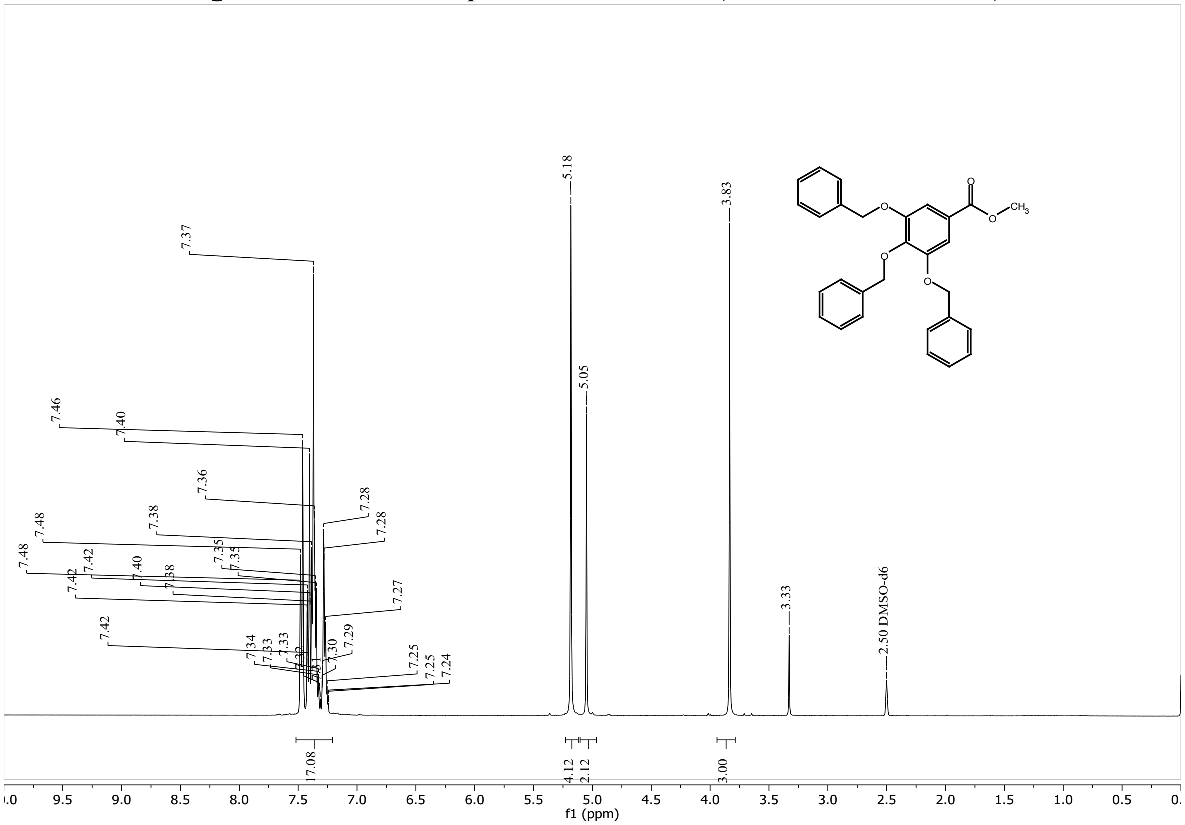

Figure S2. ^{1}H-NMR spectrum of **GA-S2** (DMSO-d_6, 400 MHz)

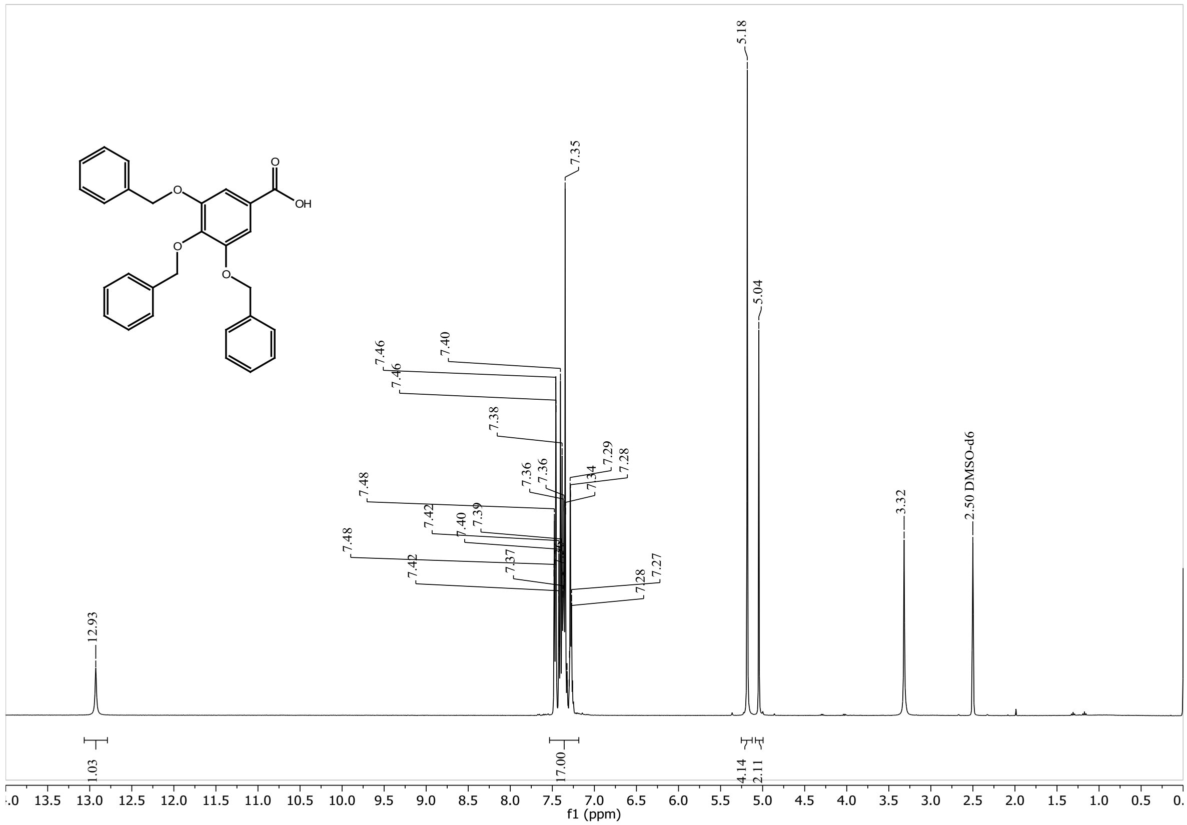

Figure S3. ^{1}H-NMR spectrum of **1** (DMSO-d_6, 400 MHz)

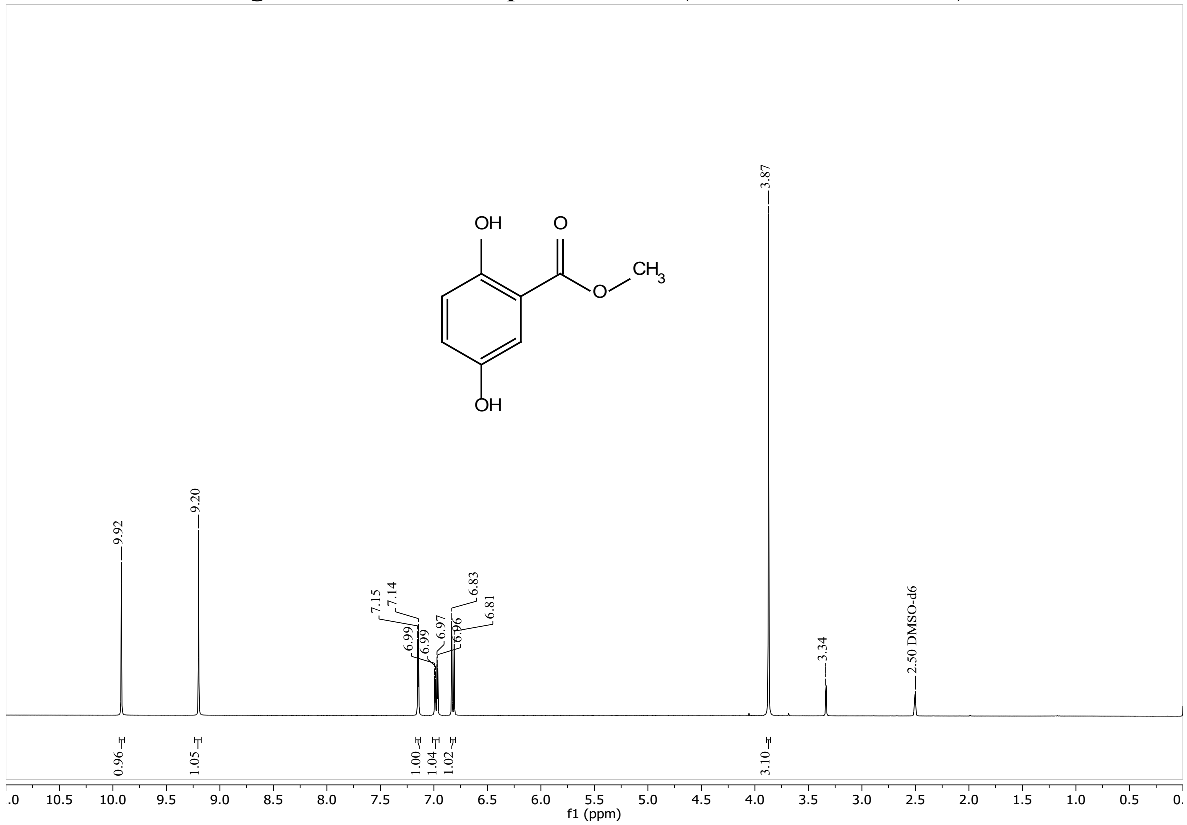

Figure S4. ^{1}H-NMR spectrum of **GE-S1** (DMSO-d_6, 400 MHz)

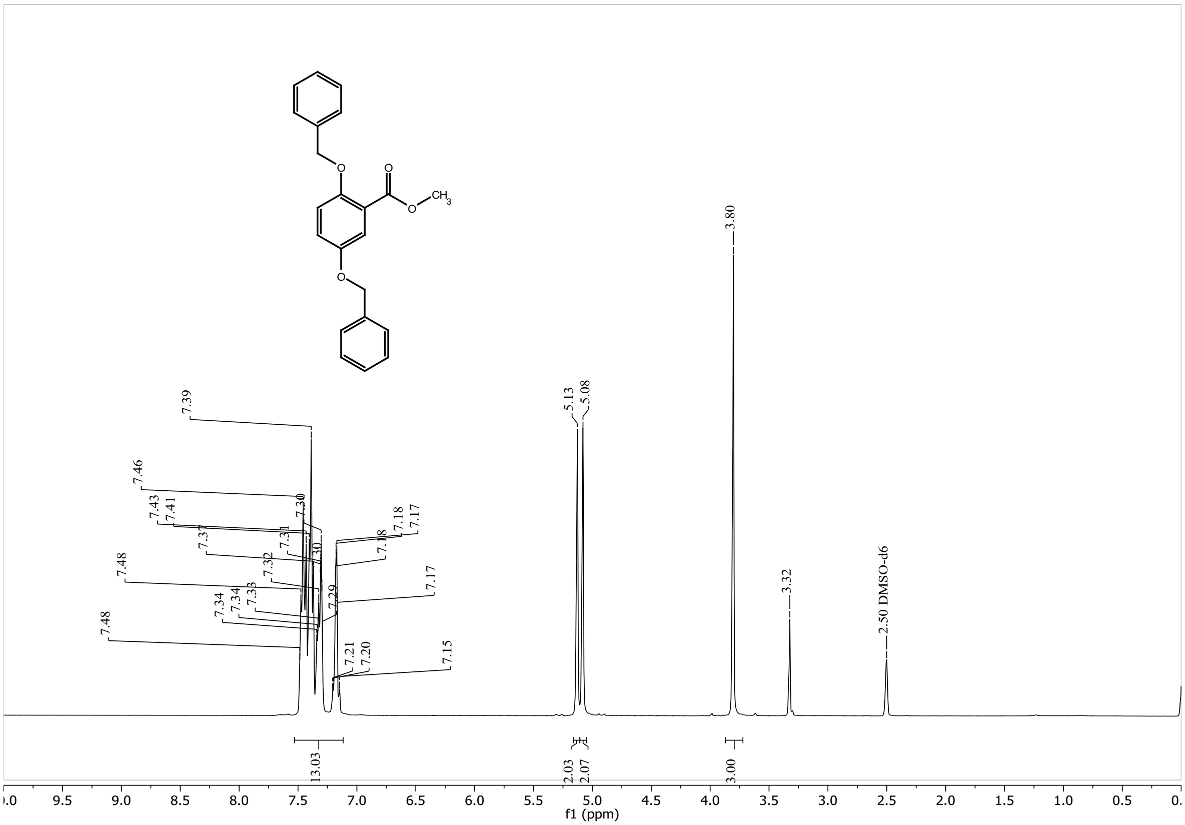

Figure S5. ^{1}H-NMR spectrum of **GE-S2** (DMSO-d_6, 400 MHz)

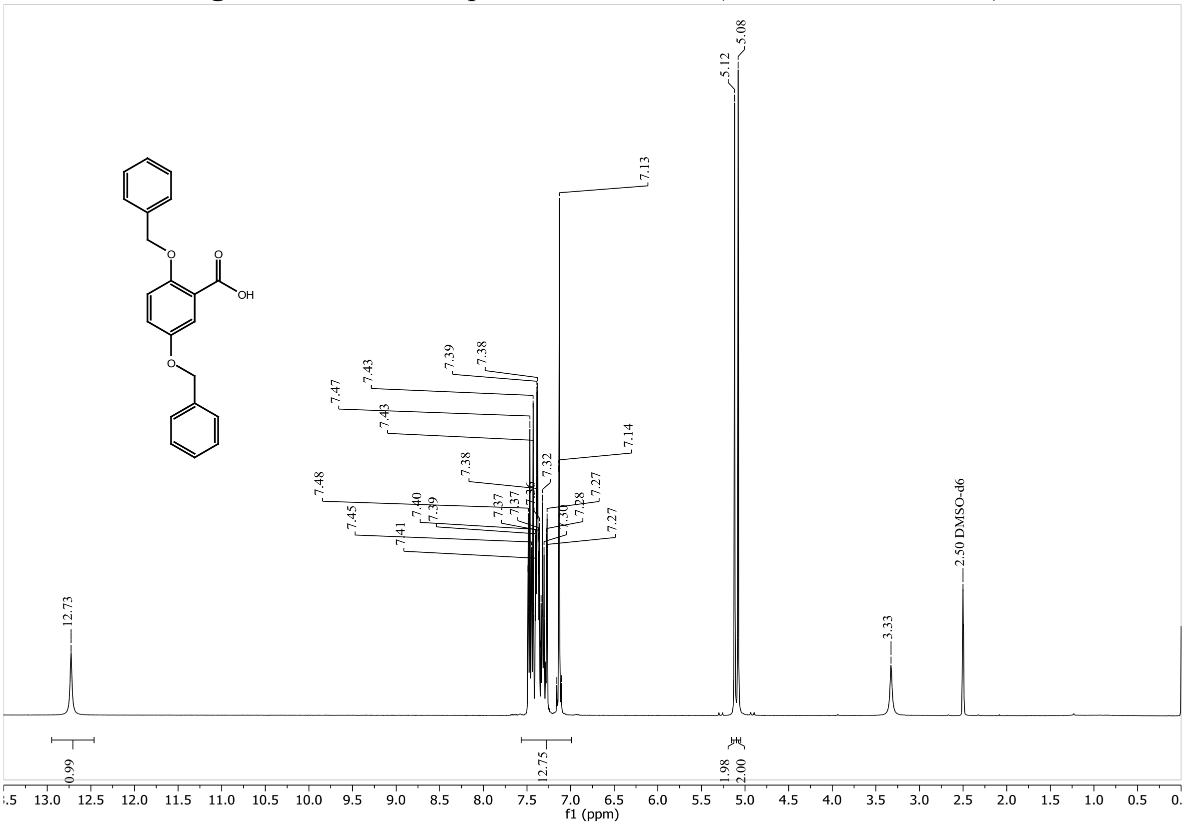

Figure S6. ^{1}H-NMR spectrum of **2** (DMSO-d_6, 400 MHz)

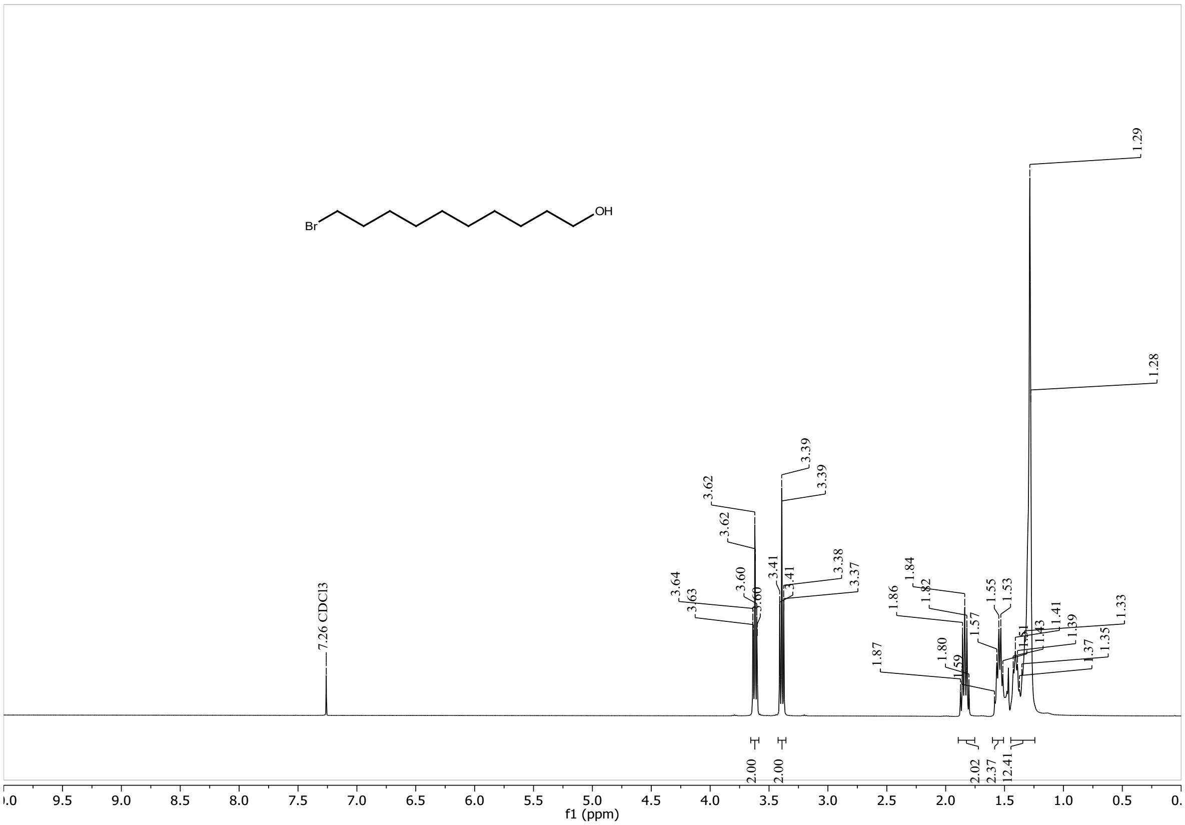

Figure S7. ^{1}H-NMR spectrum of **S1** ($CDCl_3$, 400 MHz)

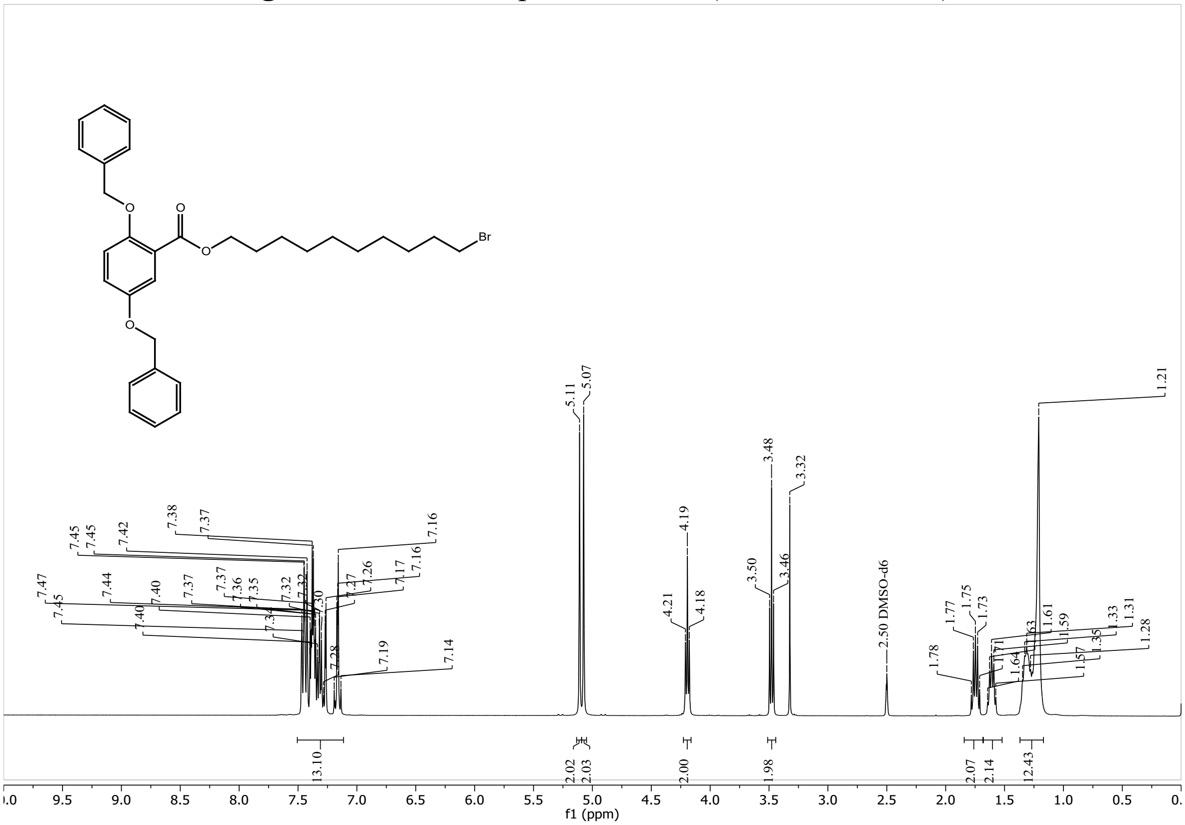

Figure S8. ^{1}H-NMR spectrum of **3** (DMSO-d_6, 400 MHz)

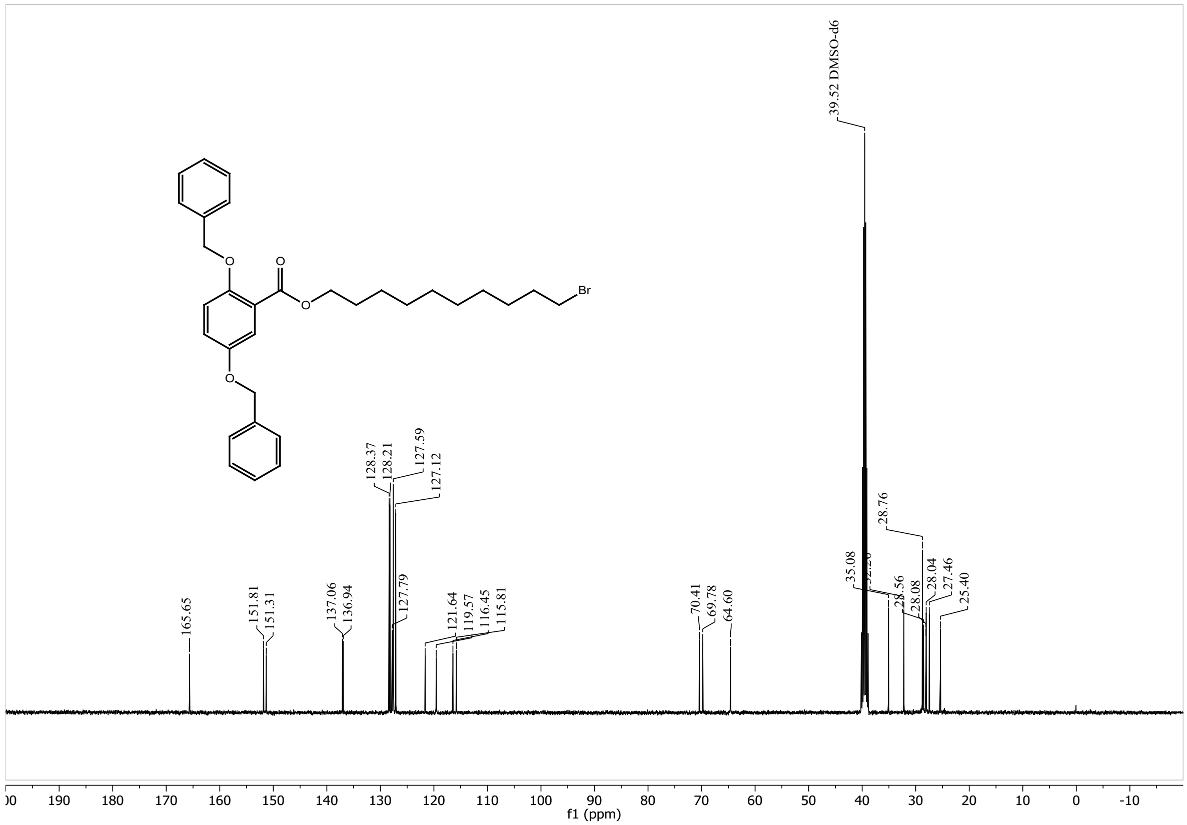

Figure S9. ^{13}C-NMR spectrum of **3** (DMSO-d_6, 101 MHz)

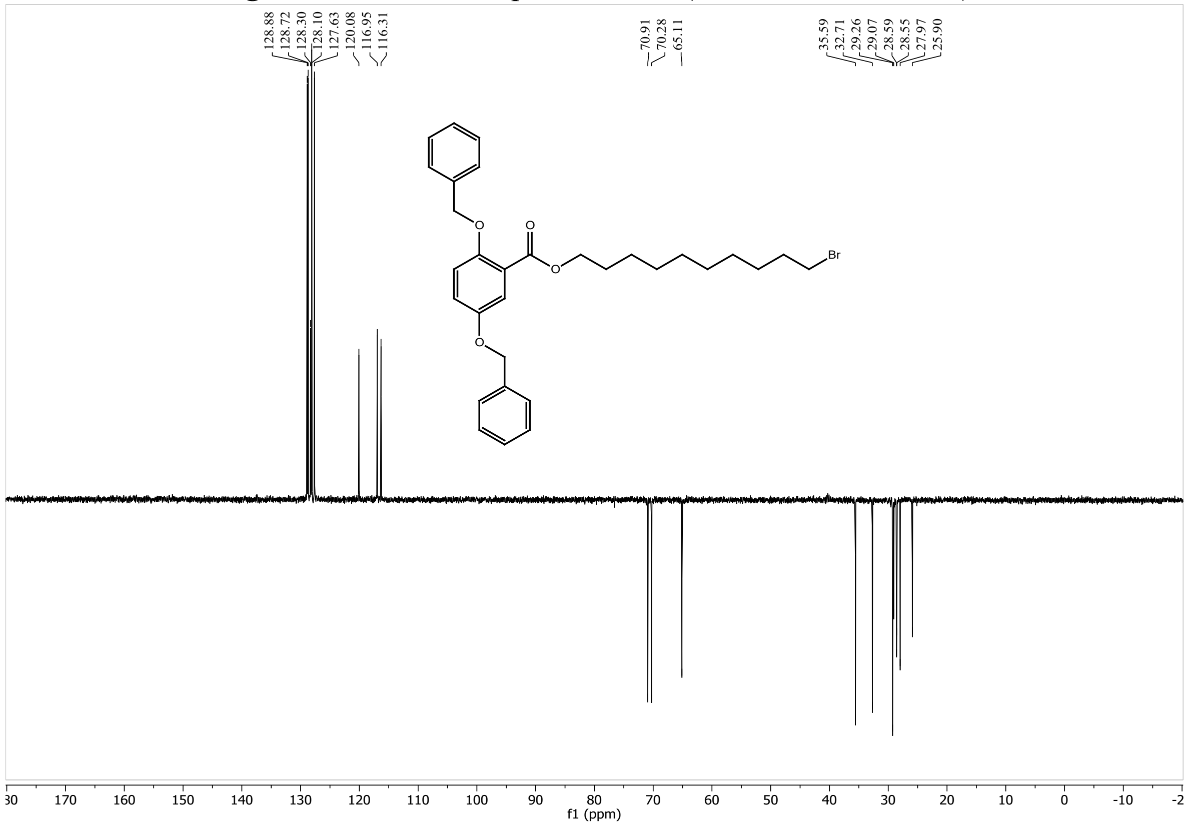

Figure S10. DEPT-135 spectrum of **3** (DMSO-d_6, 101 MHz)

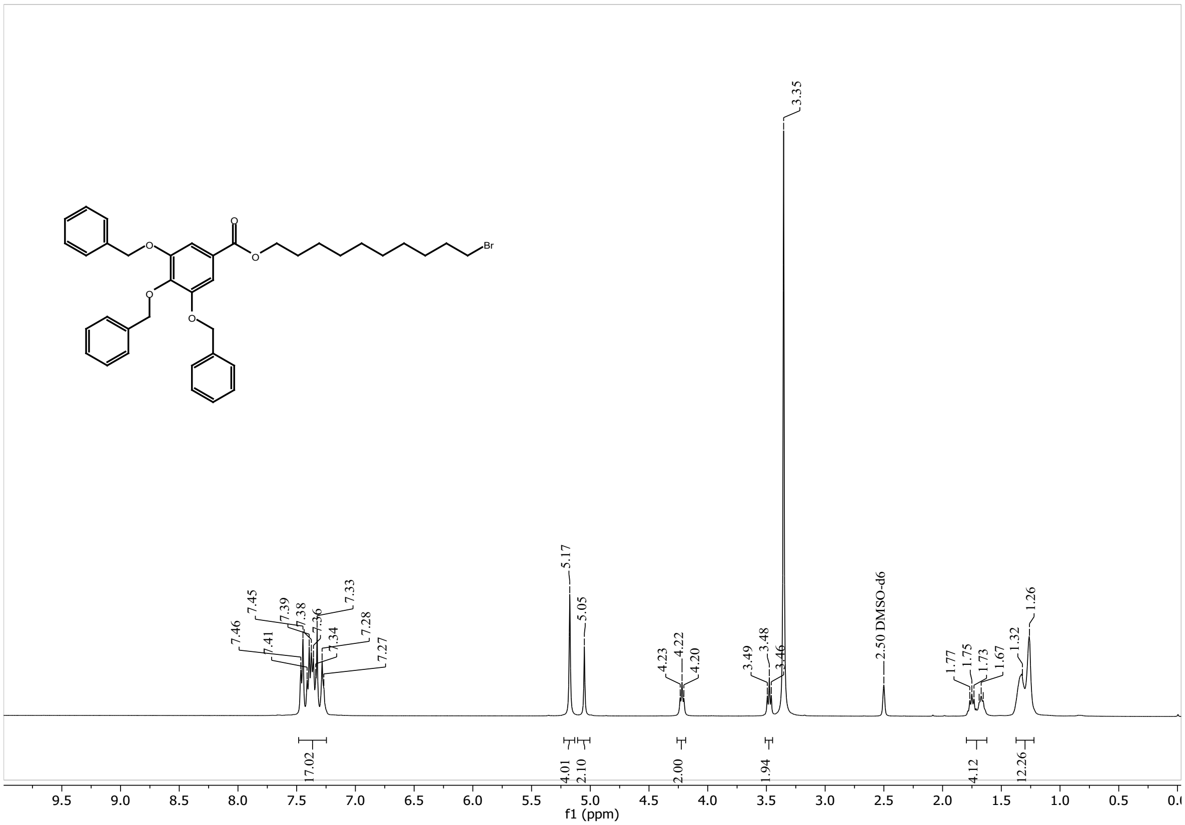

Figure S11. ^{1}H-NMR spectrum of **4** (DMSO-d_6, 400 MHz)

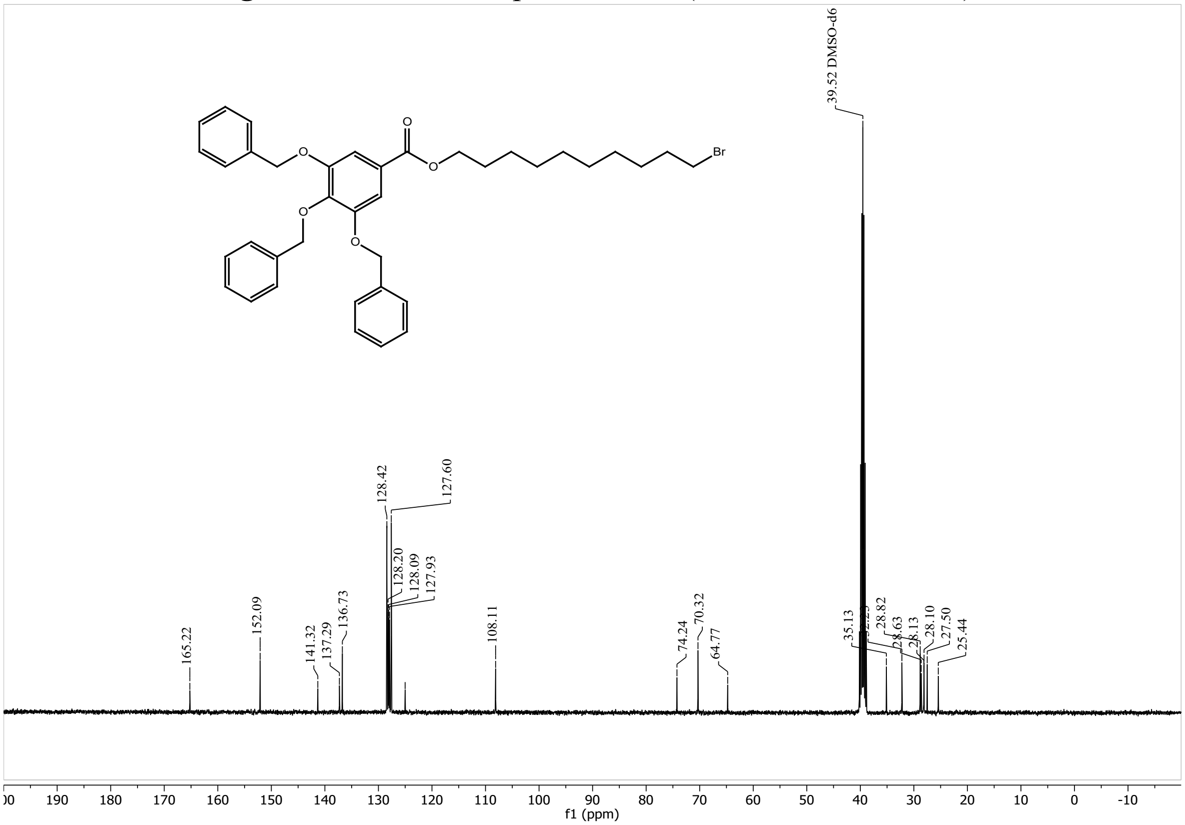

Figure S12. ^{13}C-NMR spectrum of **4** (DMSO-d_6, 101 MHz)

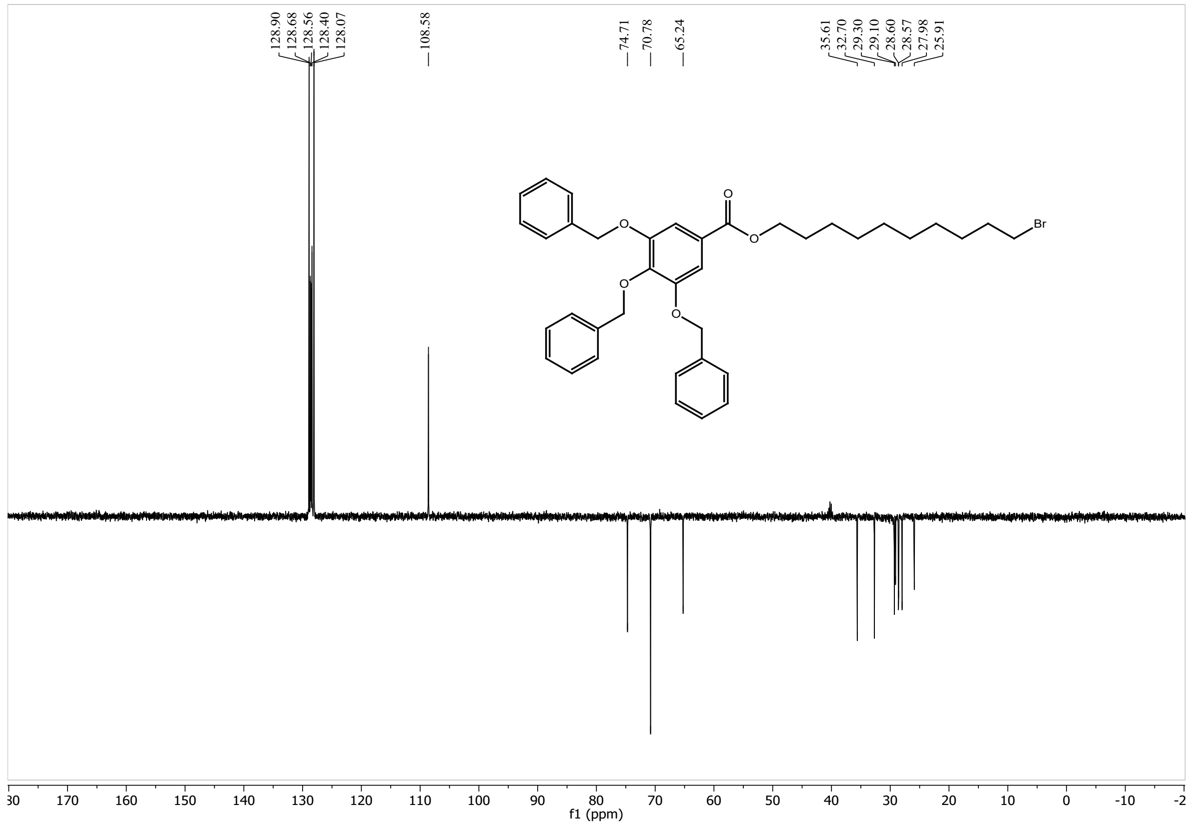

Figure S13. DEPT-135 spectrum of **4** (DMSO-d_6, 101 MHz)

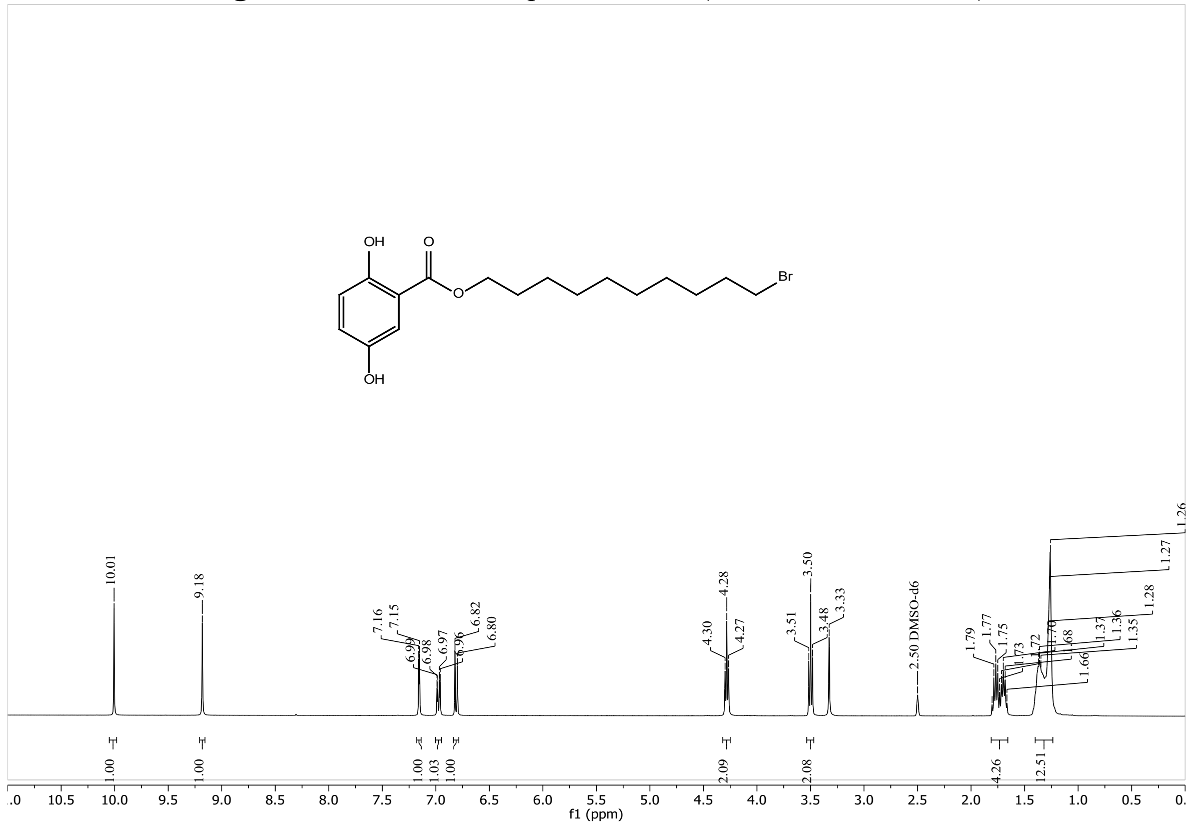

Figure S14. ^{1}H-NMR spectrum of **5** (DMSO-d_6, 400 MHz)

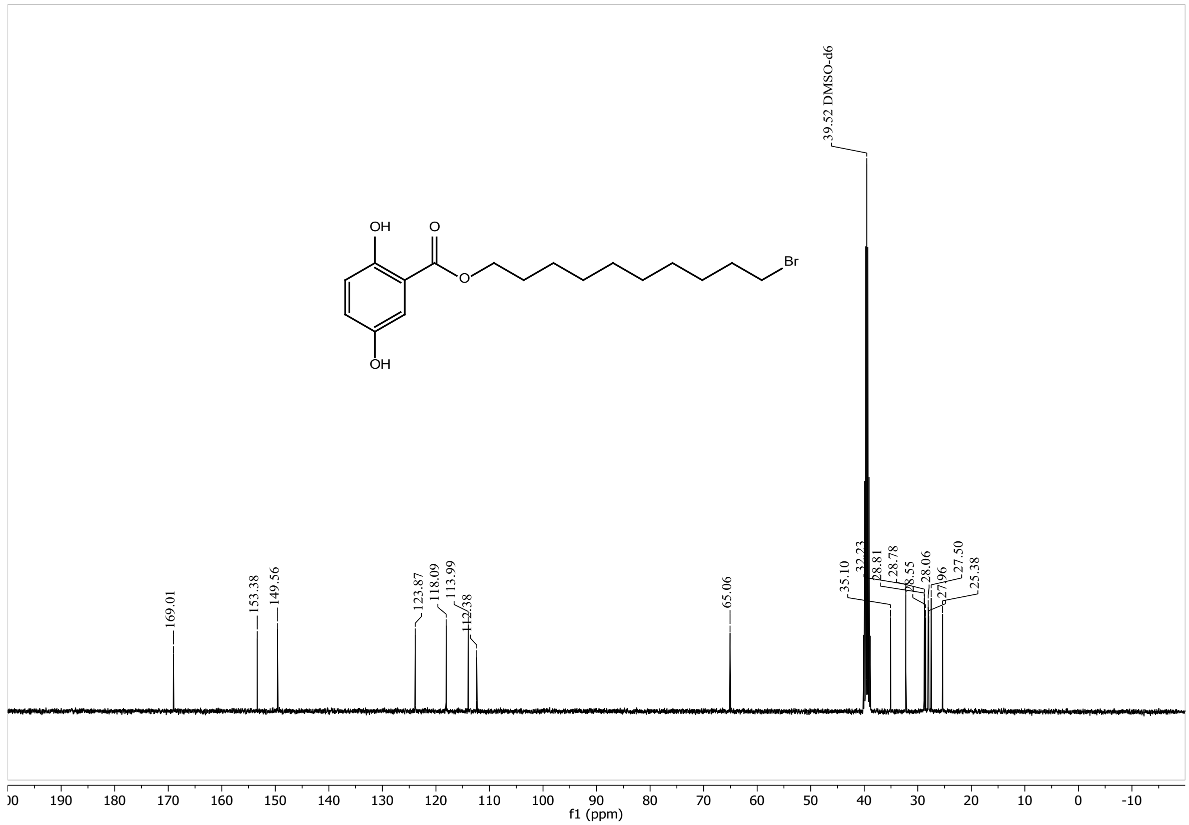

Figure S15. ^{13}C-NMR spectrum of **5** (DMSO-d_6, 101 MHz)

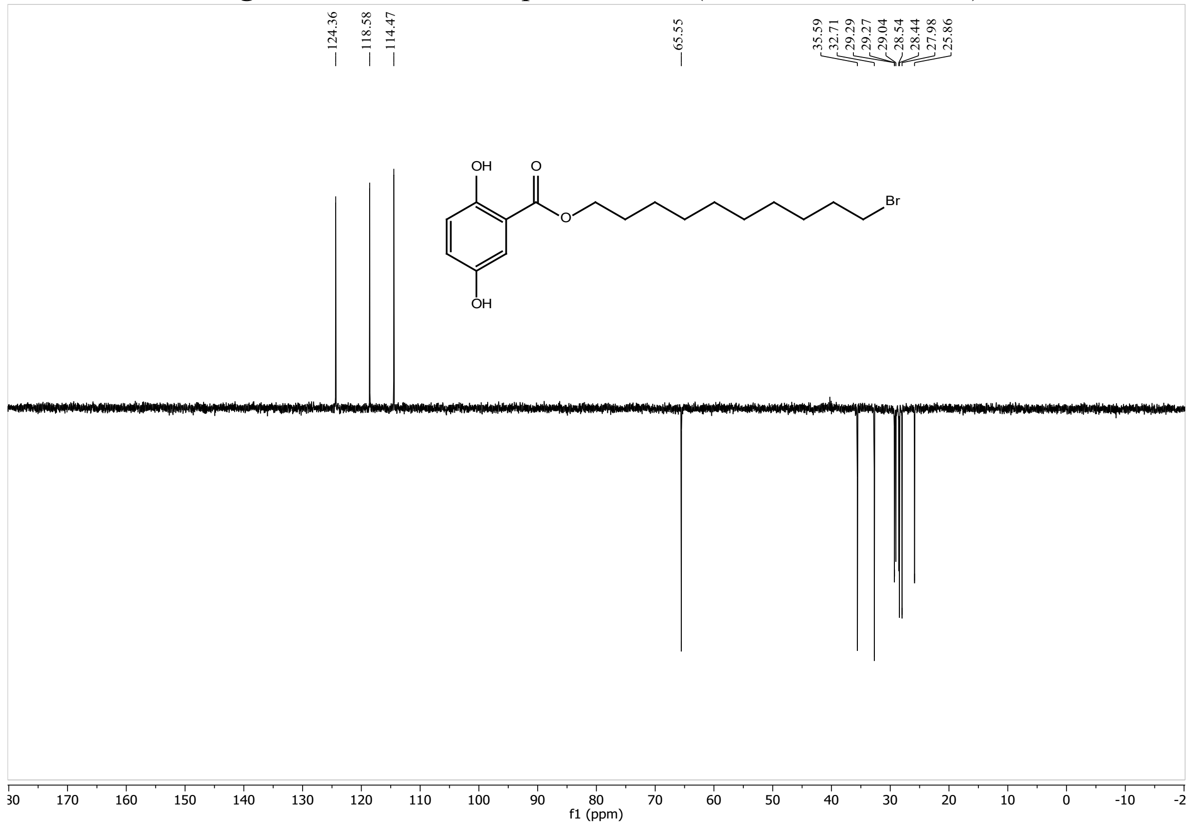

Figure S16. DEPT-135 spectrum of **5** (DMSO-d_6, 101 MHz)

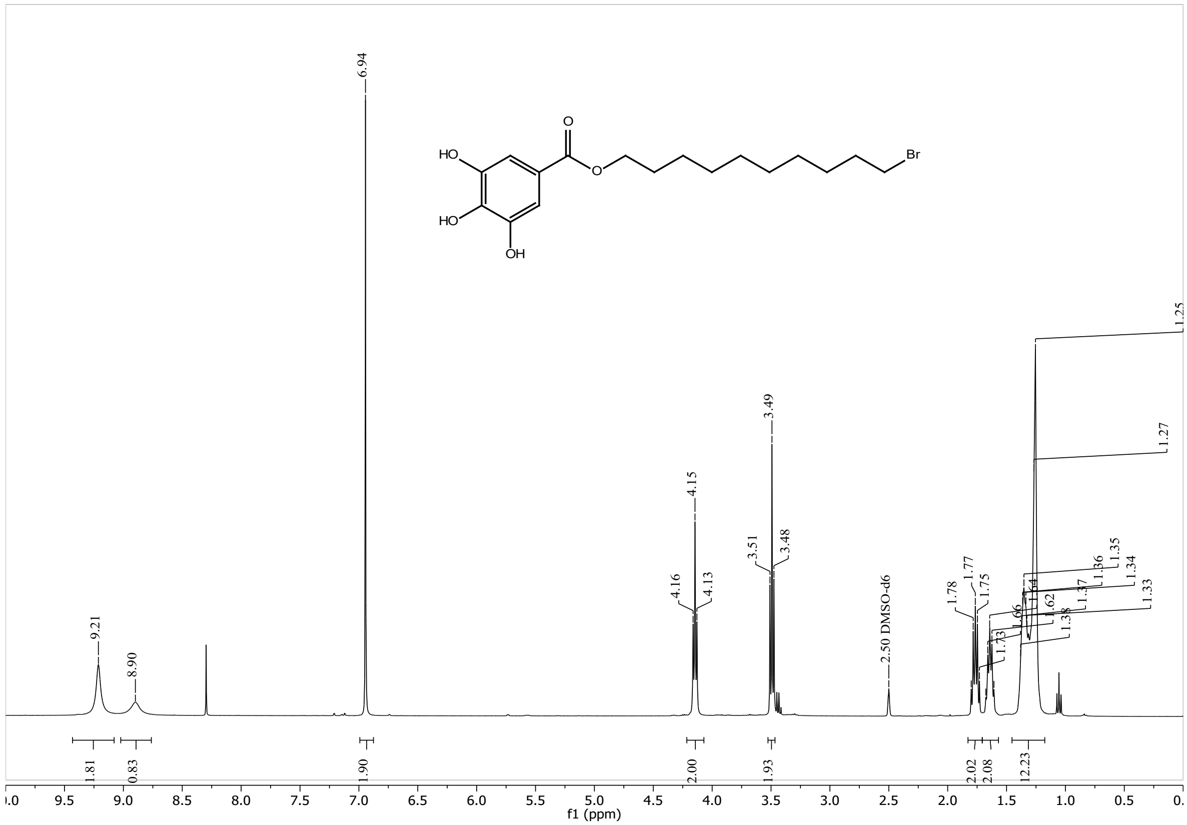

Figure S17. ^{1}H-NMR spectrum of **6** (DMSO-d_6, 400 MHz)

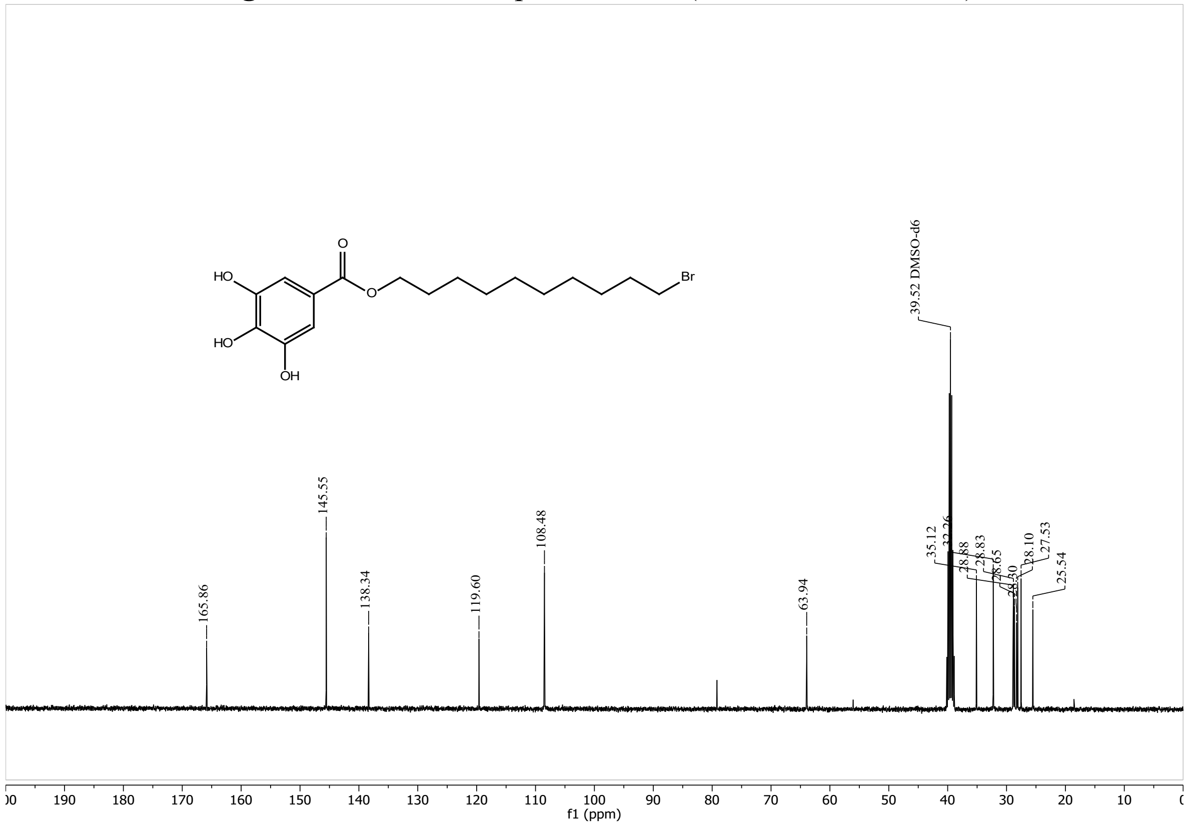

Figure S18. ^{13}C-NMR spectrum of **6** (DMSO-d_6, 101 MHz)

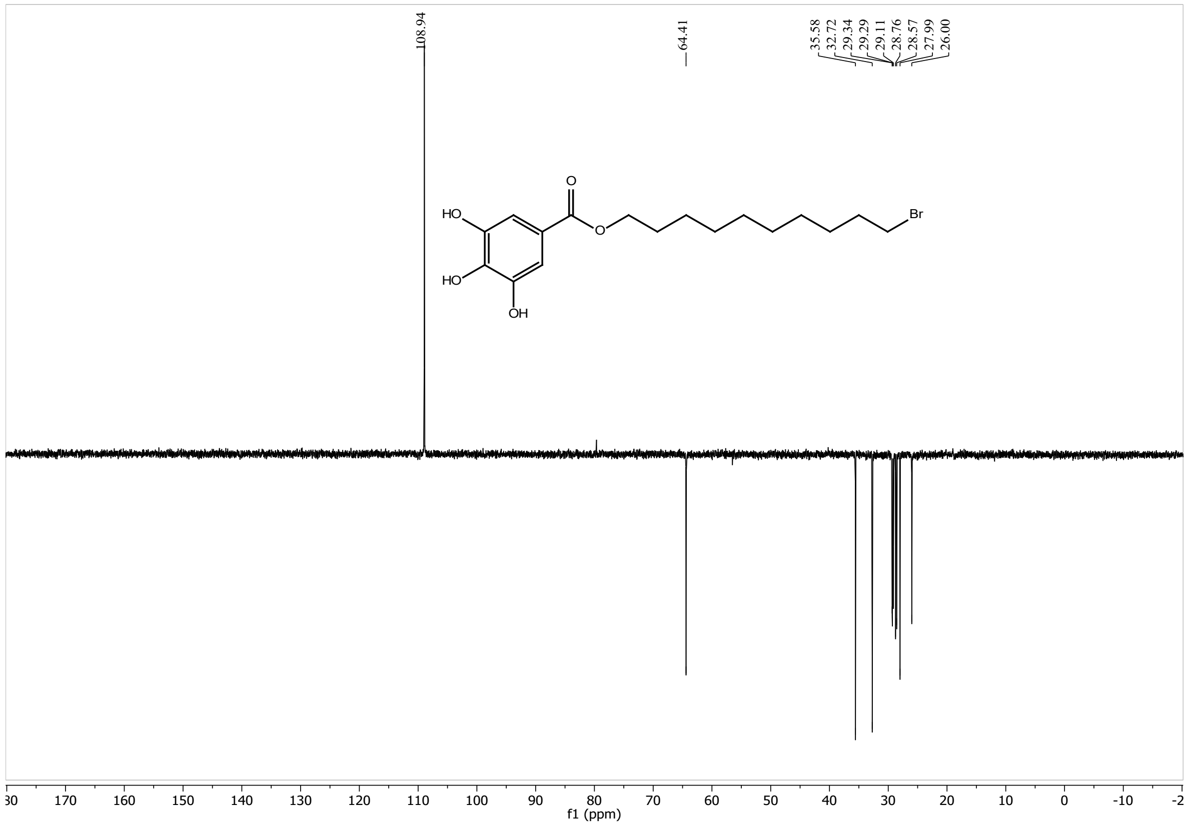

Figure S19. DEPT-135 spectrum of **6** (DMSO-d_6, 101 MHz)

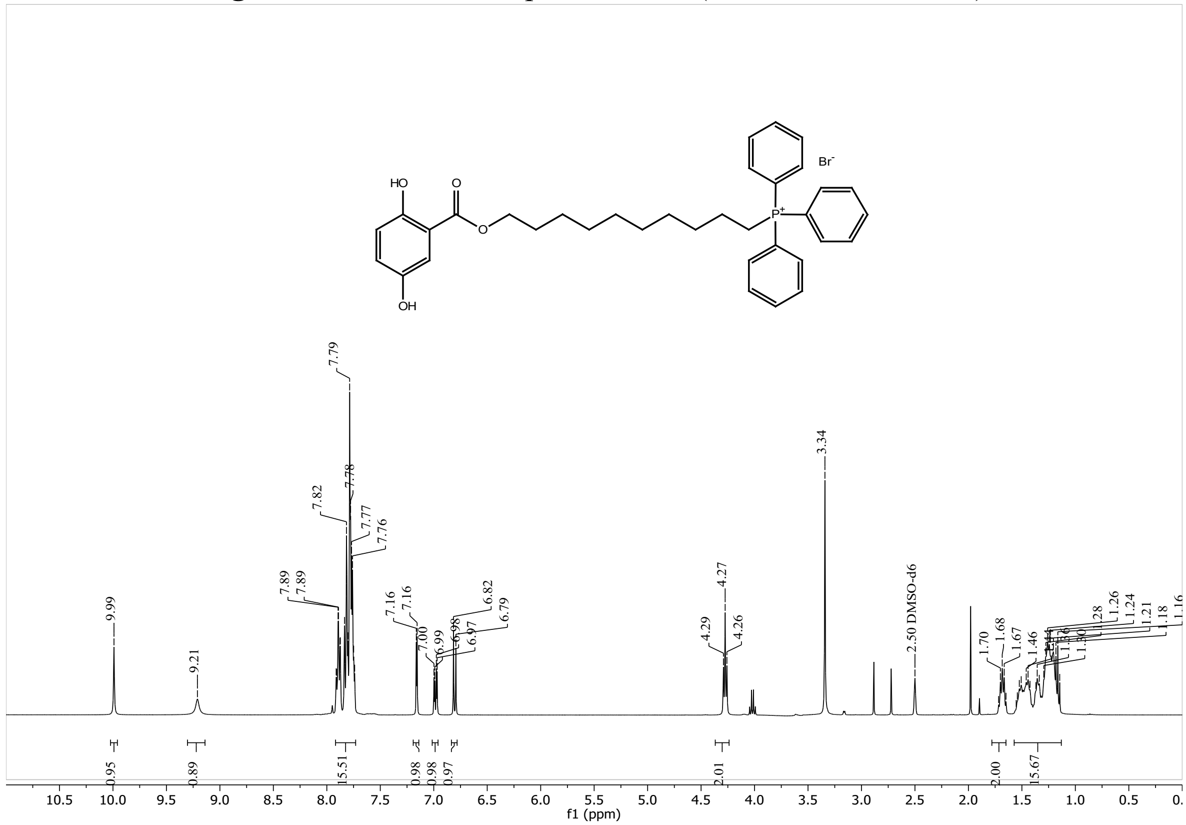

Figure S20. ^{1}H-NMR spectrum of **GE-C10-TPP**$^+$ (DMSO-d_6, 400 MHz)

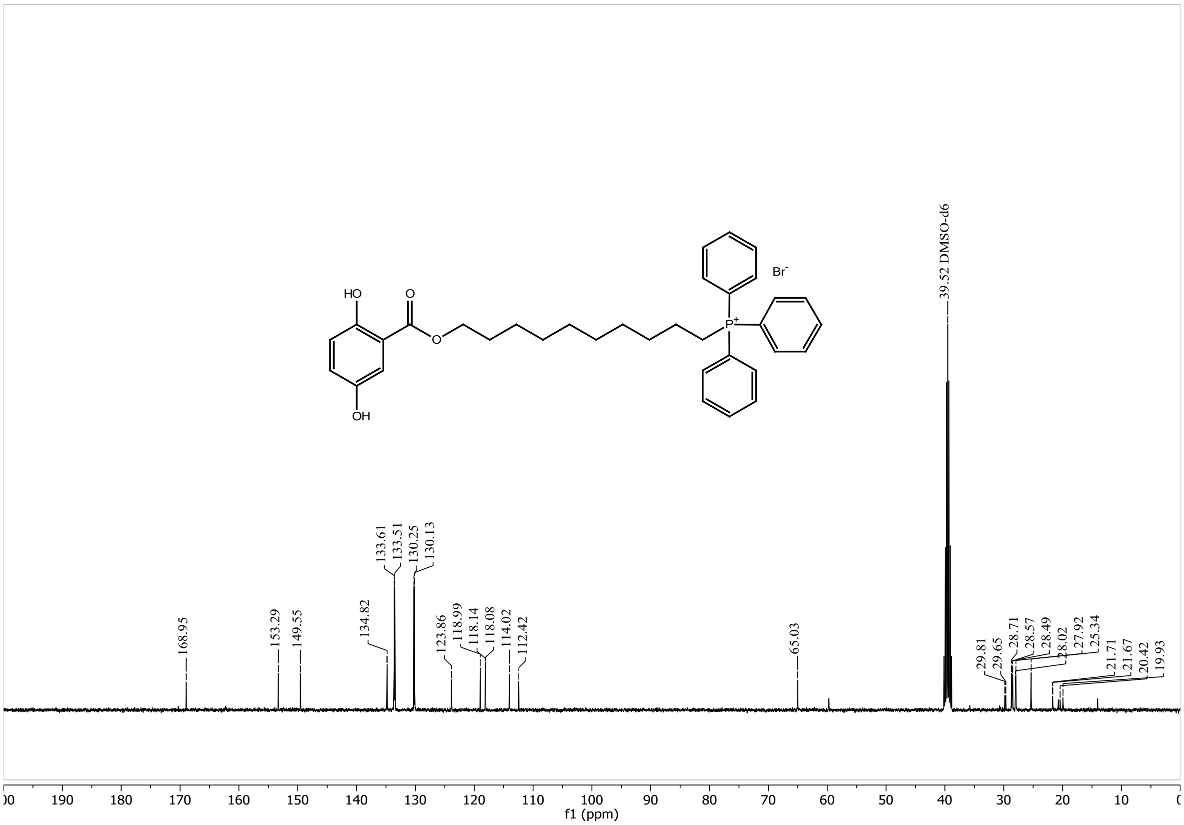

Figure S21. ^{13}C-NMR spectrum of **GE-C$_{10}$-TPP$^+$** (DMSO-d_6, 101 MHz)

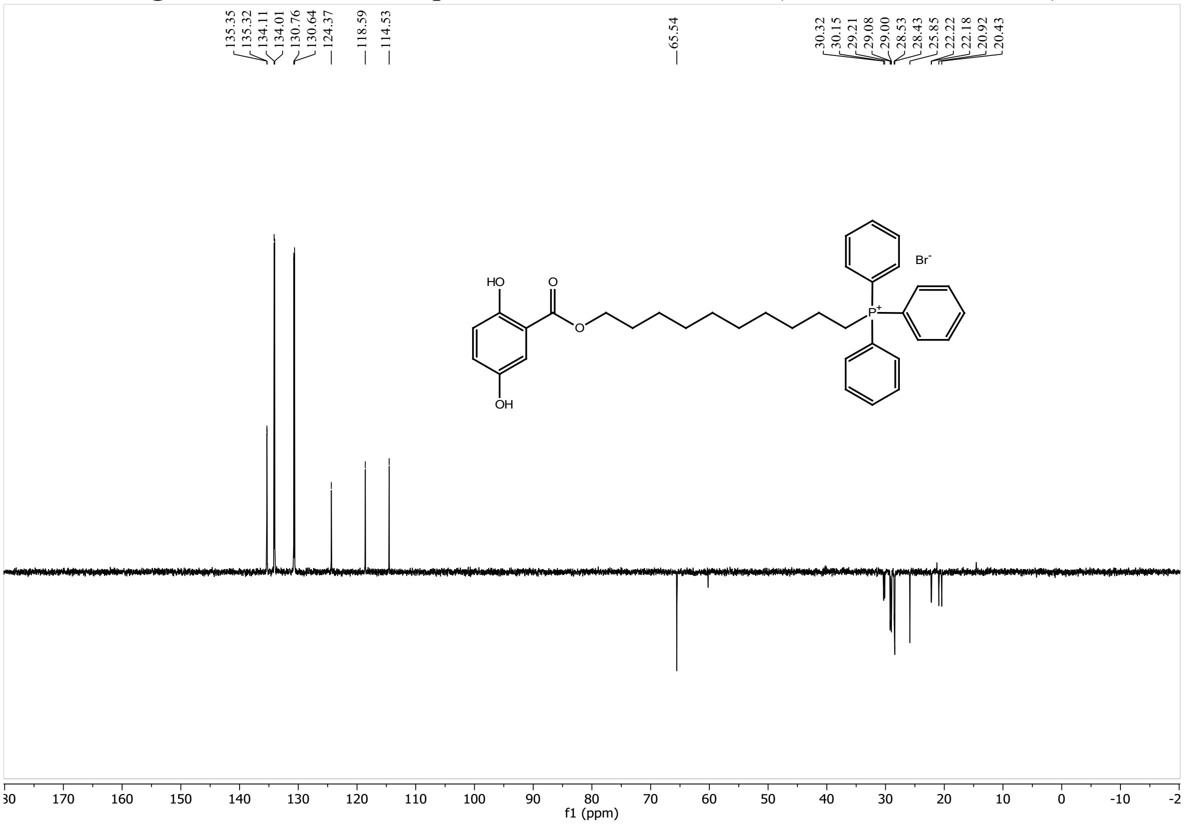

Figure S22. DEPT-135 spectrum of **GE-C$_{10}$-TPP$^+$** (DMSO-d_6, 101 MHz)

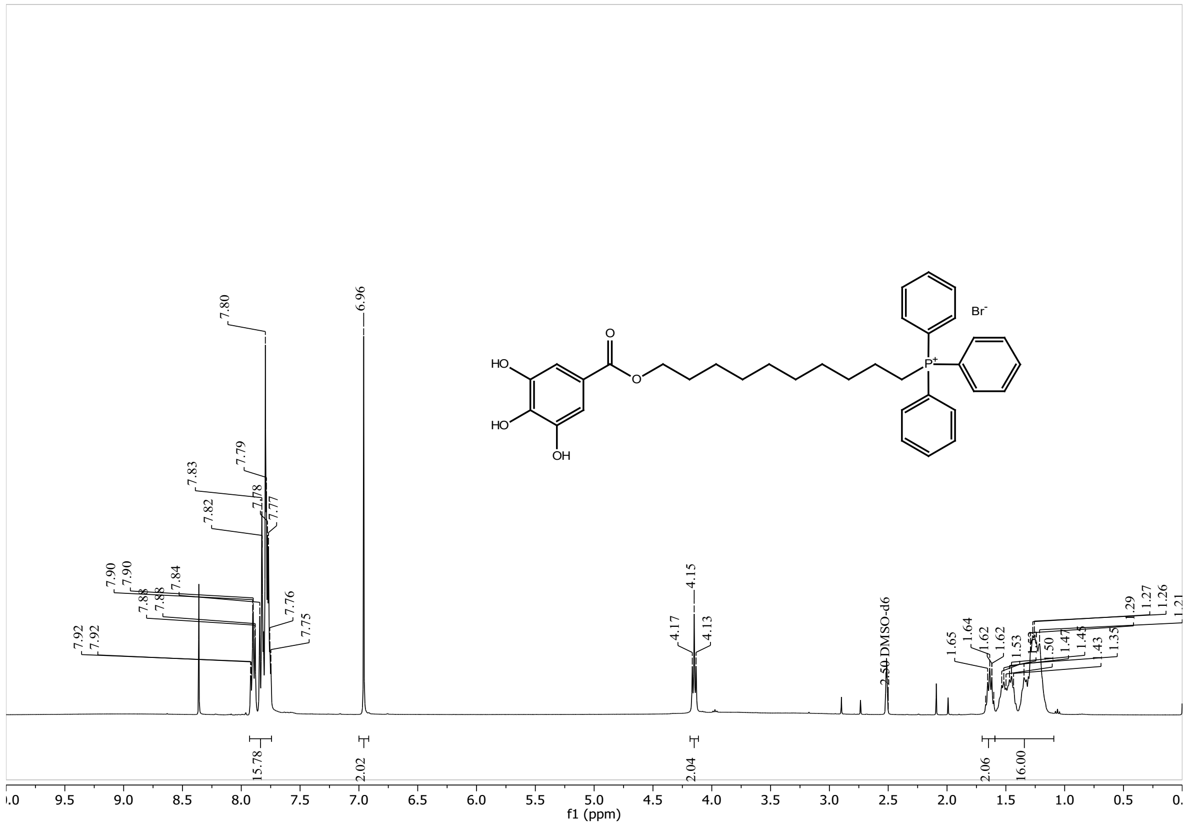

Figure S23. ^{1}H-NMR spectrum of **GA-C10-TPP^{+}** (DMSO-d_6, 400 MHz)

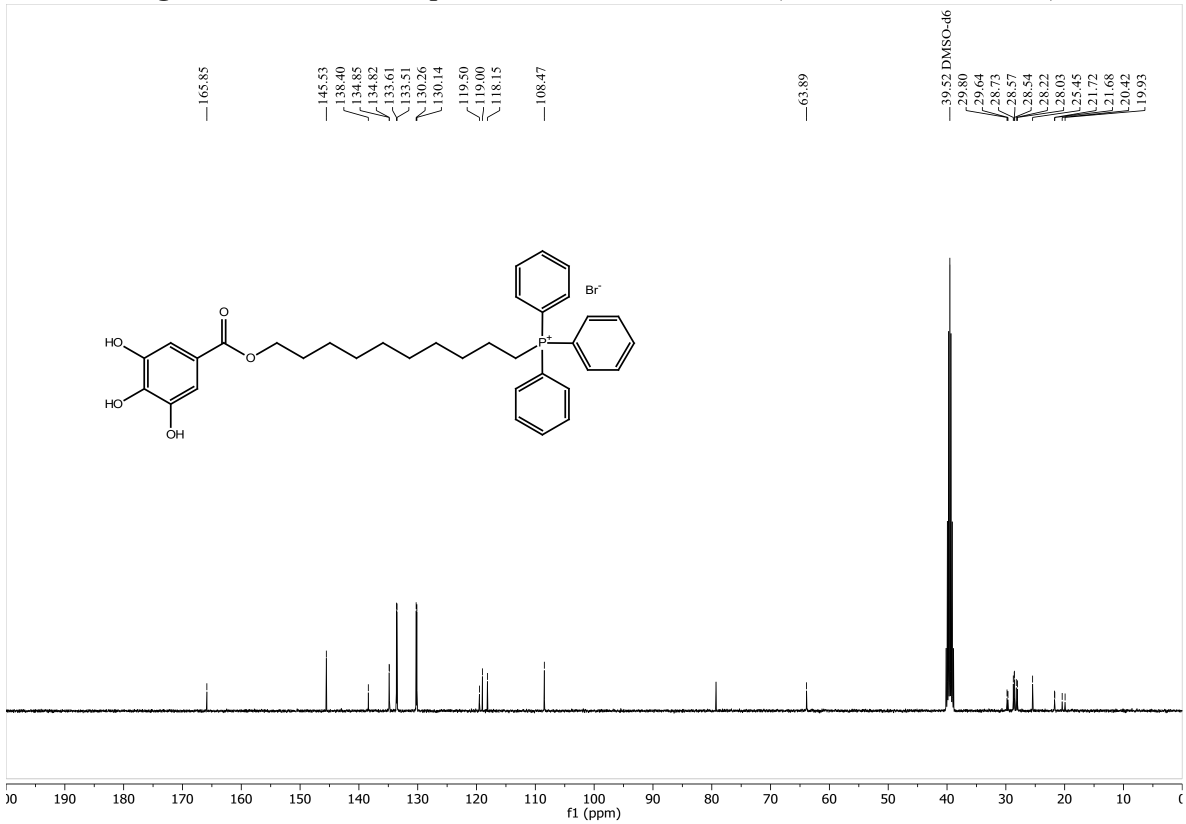

Figure S24. ^{13}C-NMR spectrum of **GA-C10-TPP^{+}** (DMSO-d_6, 101 MHz)

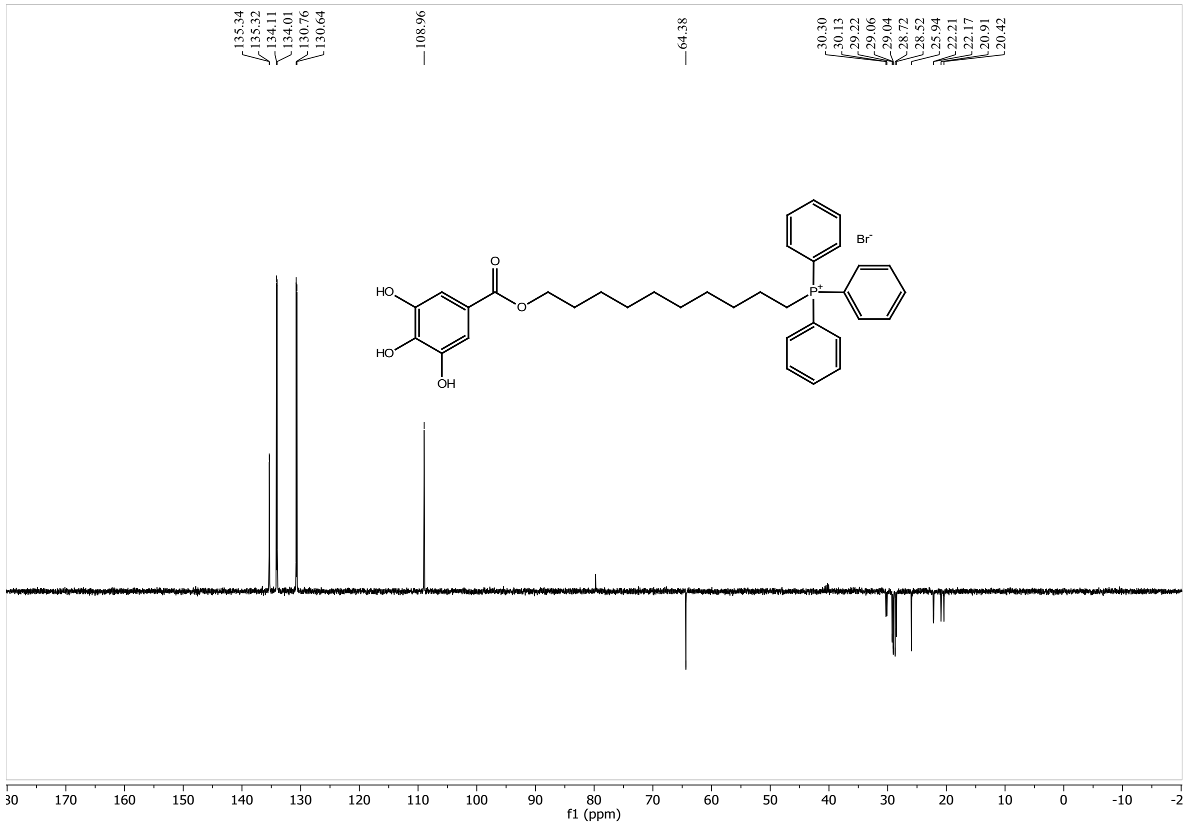

Figure S25. DEPT-135 spectrum of **GA-C10-TPP**$^{+}$ (DMSO-d_6, 101 MHz)
