# Supplementary material for: Antimigratory Effect of Lipophilic Cations Derived from Gallic and Gentisic Acid and Synergistic Effect with 5-Fluorouracil on Metastatic Colorectal Cancer Cells: A New Synthesis Route
Source: Cancers (Basel). 2024 Aug 27;16(17):2980. doi: 10.3390/cancers16172980 (PMC11393949; doi:10.3390/cancers16172980)

Representative blots

The standard Ladder we used in this work was Accuruler for Tris-Glycine gels

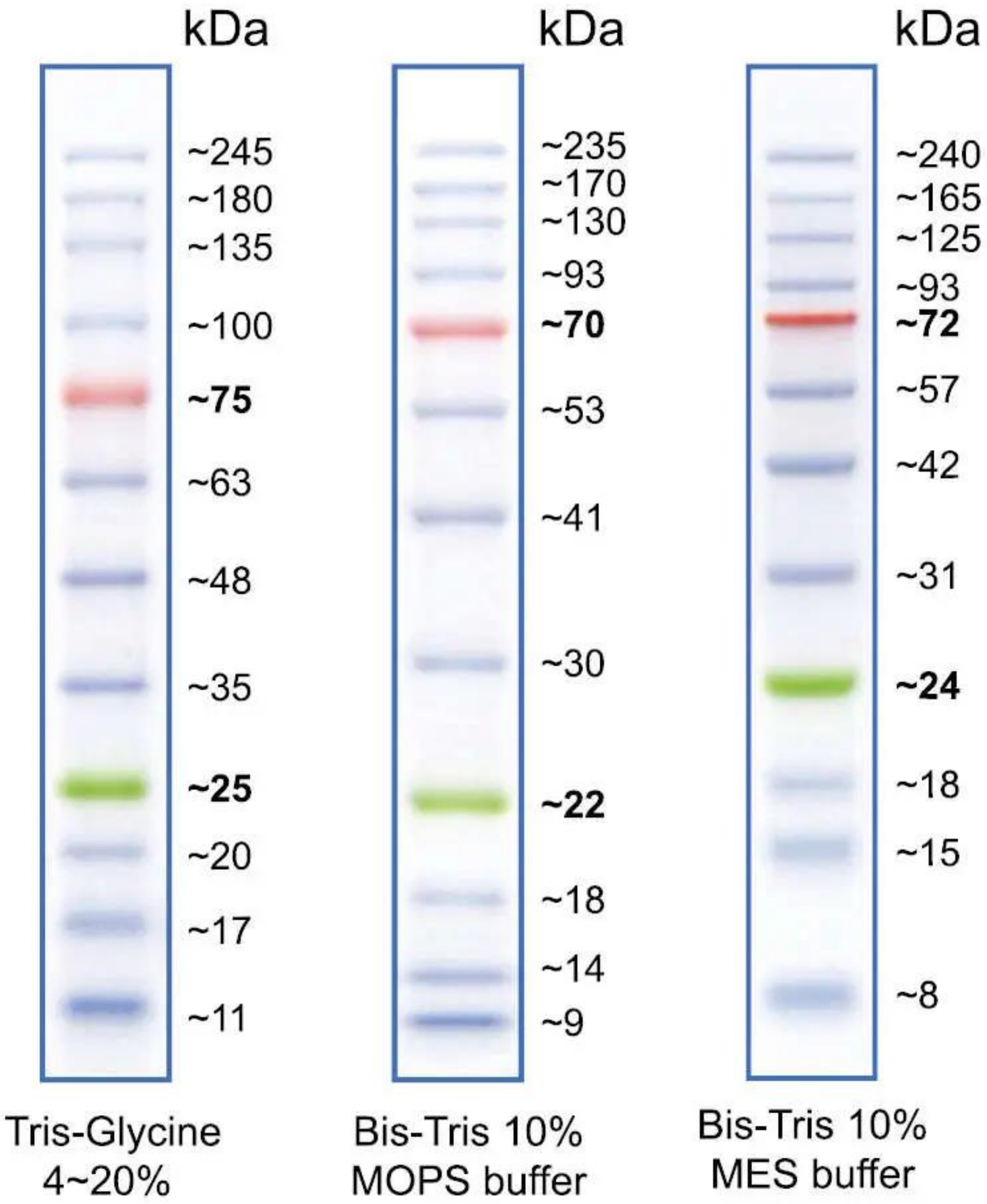

Figure 3 E

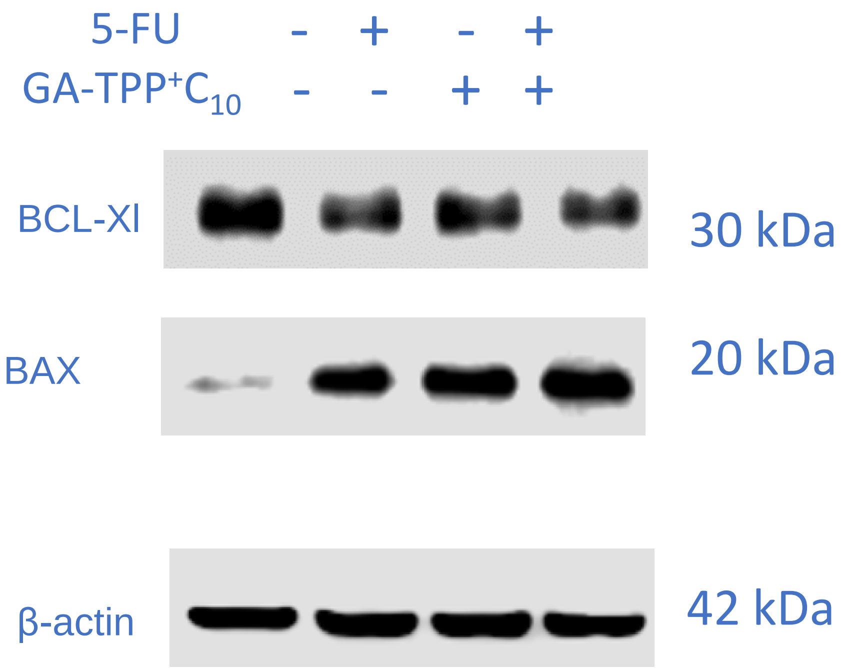

Figure 4 A

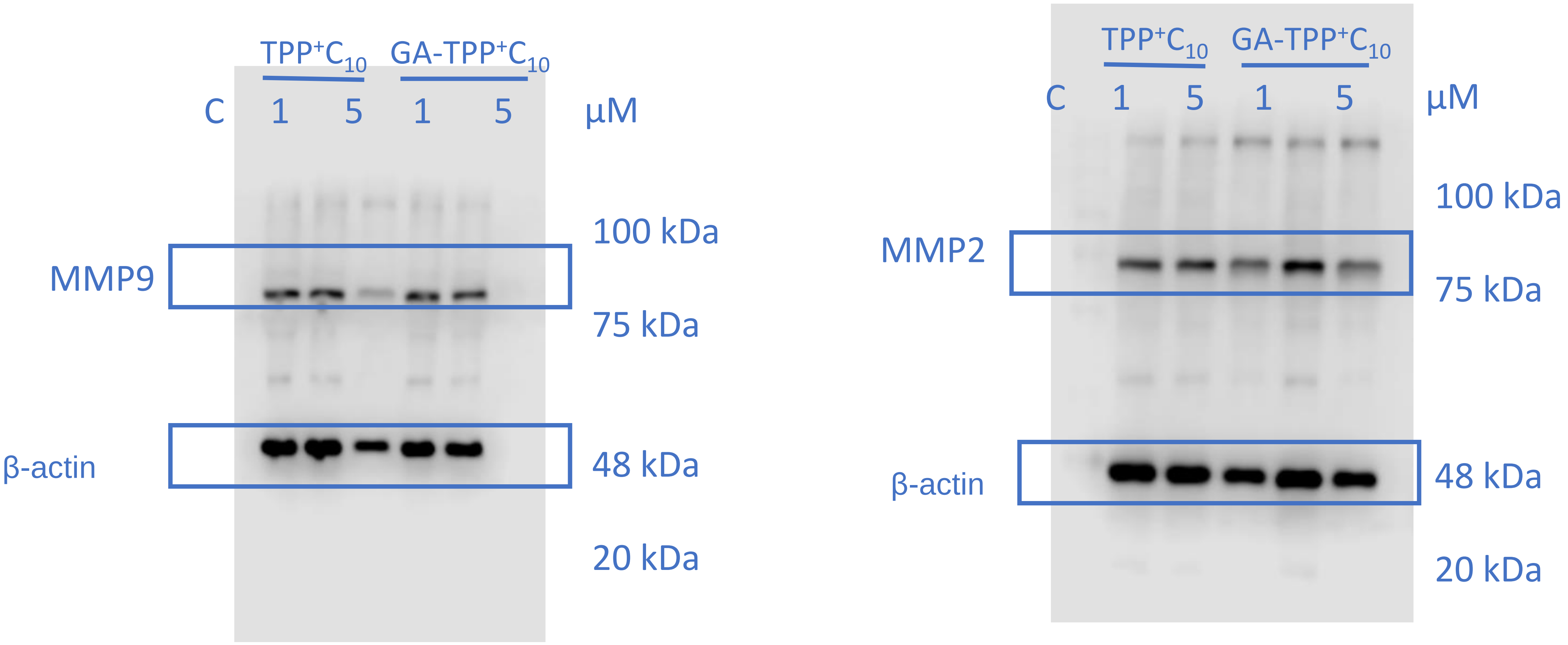
TPP⁺C10
GA-TPP⁺C10
C
1
5
1
5
µM
100 kDa
MMP9
75 kDa
β-actin
48 kDa
20 kDa
TPP⁺C10
GA-TPP⁺C10
C
1
5
1
5
µM
100 kDa
MMP2
75 kDa
β-actin
48 kDa
20 kDa

Figure 4 F

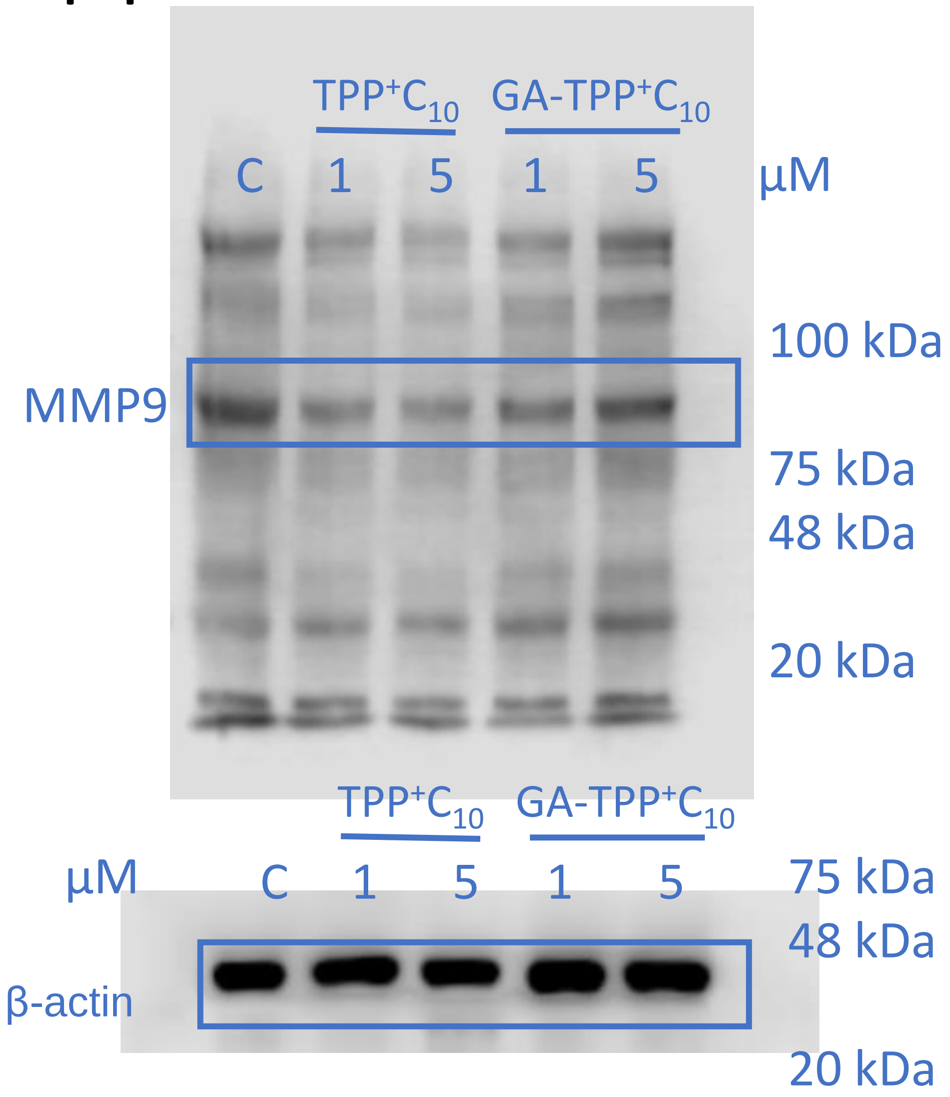

TPP+C10
GA-TPP+C10
C 1 5 1 5 µM
100 kDa
MMP9
75 kDa
48 kDa
20 kDa
TPP+C10
GA-TPP+C10
µM C 1 5 1 5
75 kDa
48 kDa
β-actin
20 kDa

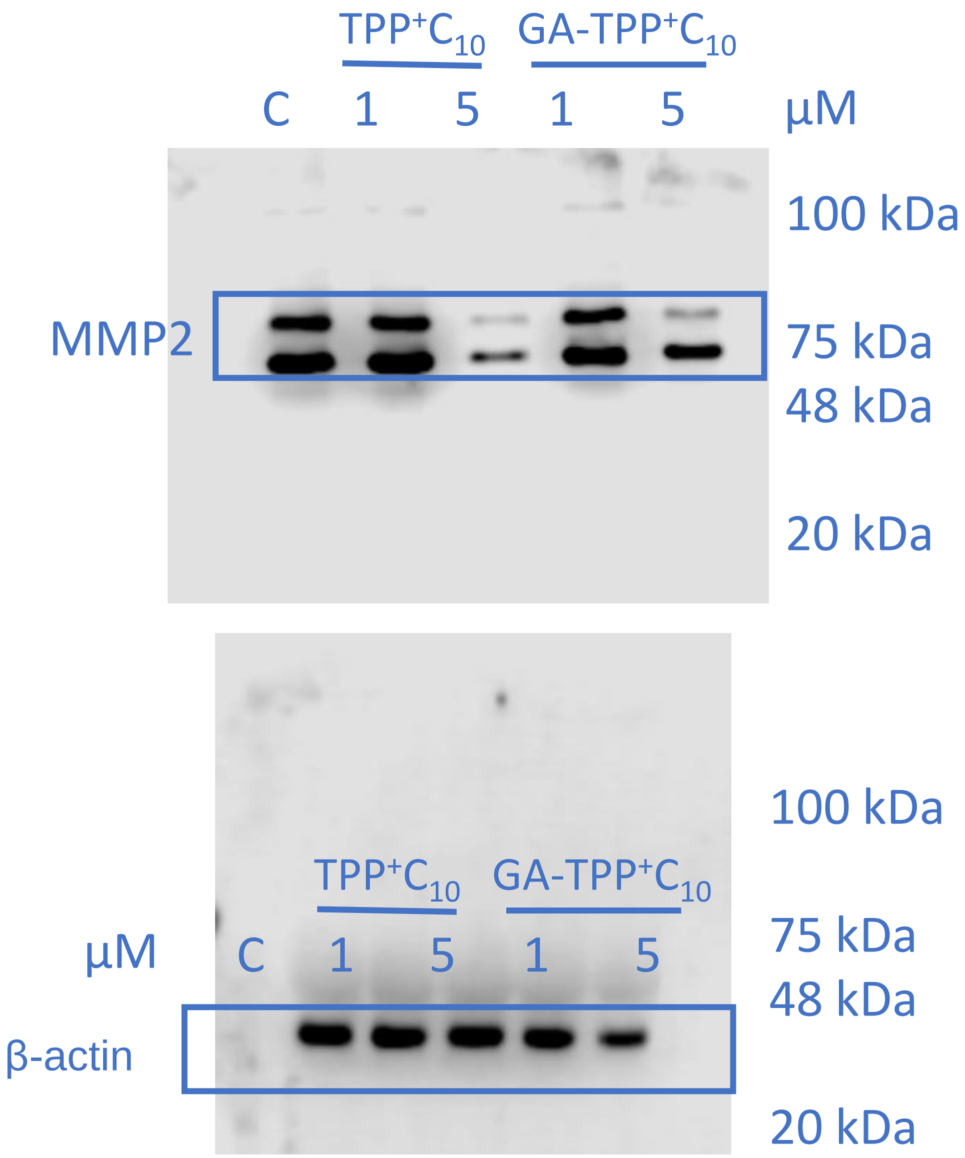

TPP+C10
GA-TPP+C10
C 1 5 1 5 µM
100 kDa
MMP2
75 kDa
48 kDa
20 kDa
100 kDa
TPP+C10
GA-TPP+C10
µM C 1 5 1 5
75 kDa
48 kDa
β-actin
20 kDa

Figure 4 K

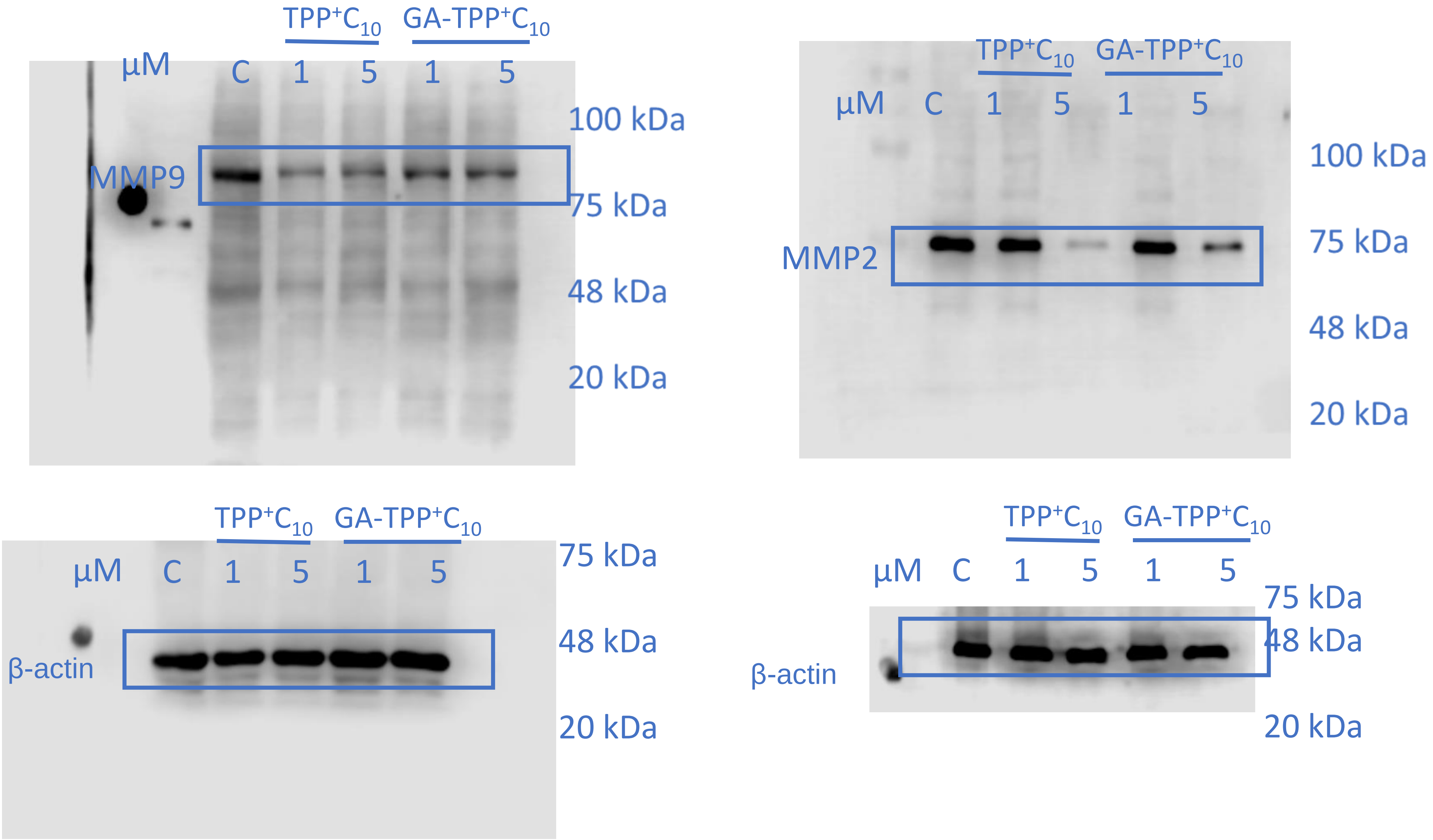
TPP+C10
GA-TPP+C10
µM
C
1
5
1
5
100 kDa
MMP9
75 kDa
48 kDa
20 kDa
TPP+C10
GA-TPP+C10
µM
C
1
5
1
5
100 kDa
MMP2
75 kDa
48 kDa
20 kDa
TPP+C10
GA-TPP+C10
µM
C
1
5
1
5
75 kDa
β-actin
48 kDa
20 kDa
TPP+C10
GA-TPP+C10
µM
C
1
5
1
5
75 kDa
48 kDa
β-actin
20 kDa

Figure 5 A

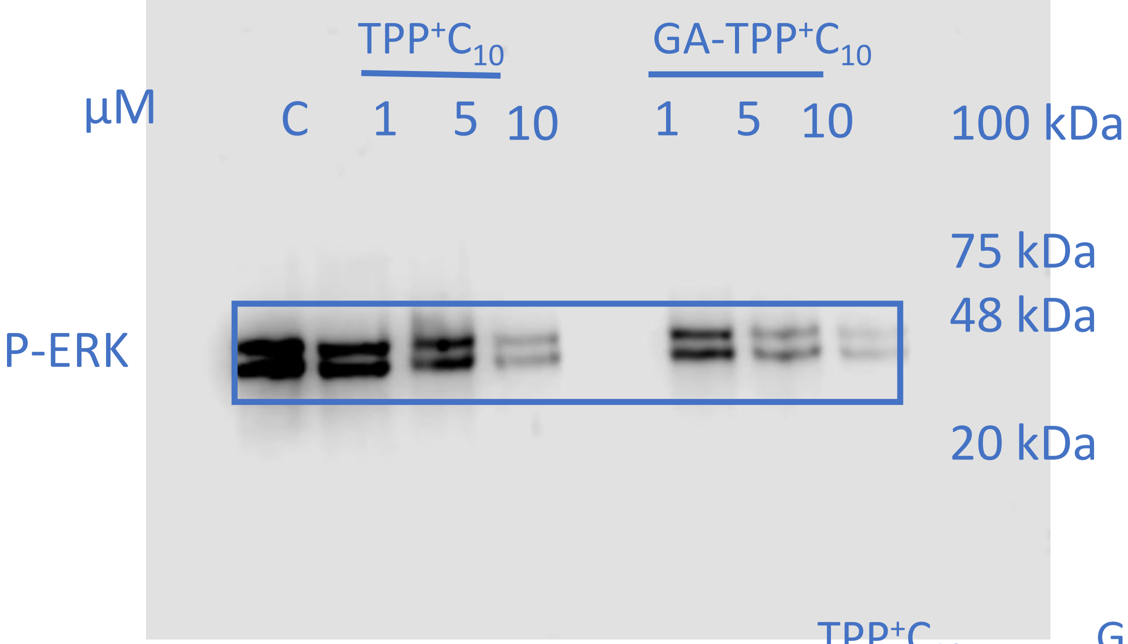

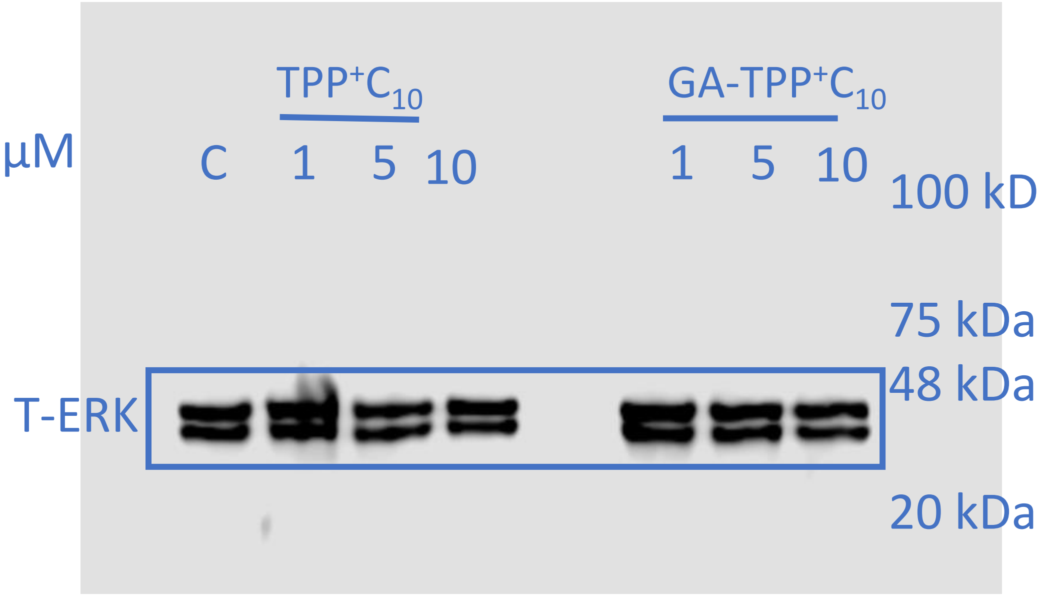

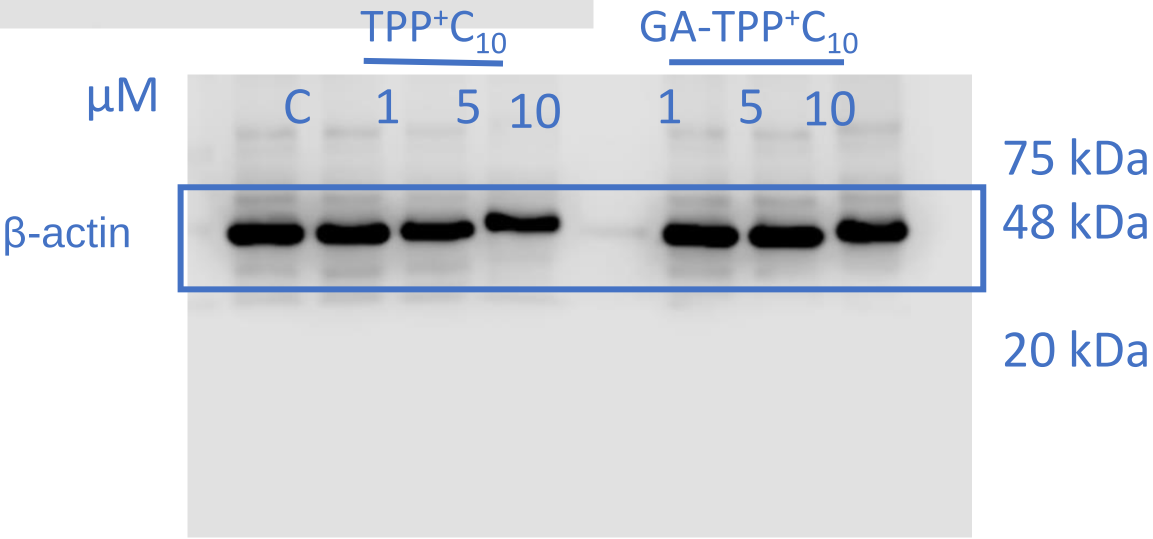

The concentration of 10 uM was not included in the final blot, since it reduce cell viability

Figure 5 A

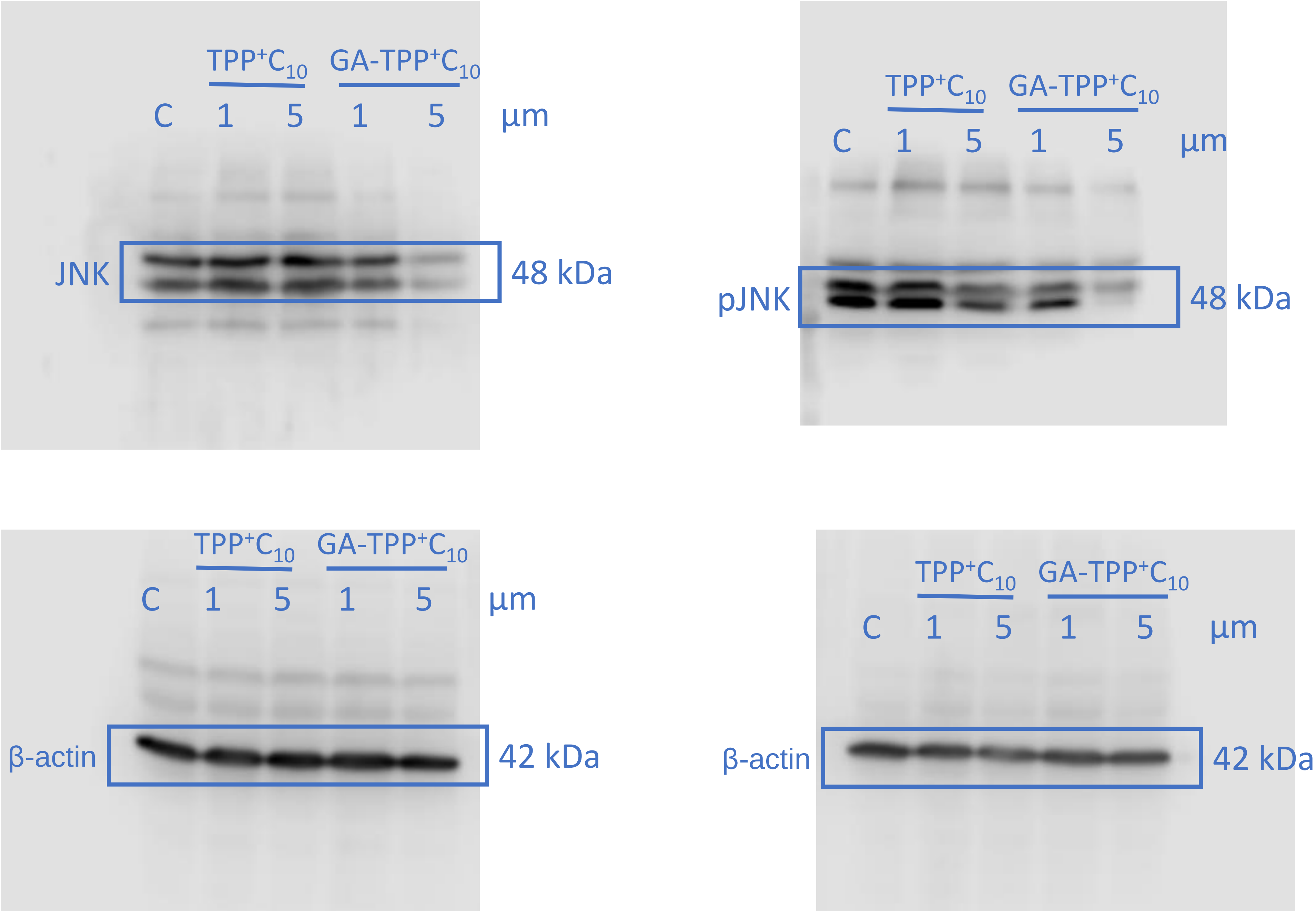
TPP+C10 GA-TPP+C10
C 1 5 1 5 µm
JNK
48 kDa
TPP+C10 GA-TPP+C10
C 1 5 1 5 µm
pJNK
48 kDa
TPP+C10 GA-TPP+C10
C 1 5 1 5 µm
β-actin
42 kDa
TPP+C10 GA-TPP+C10
C 1 5 1 5 µm
β-actin
42 kDa

Figure 5 A

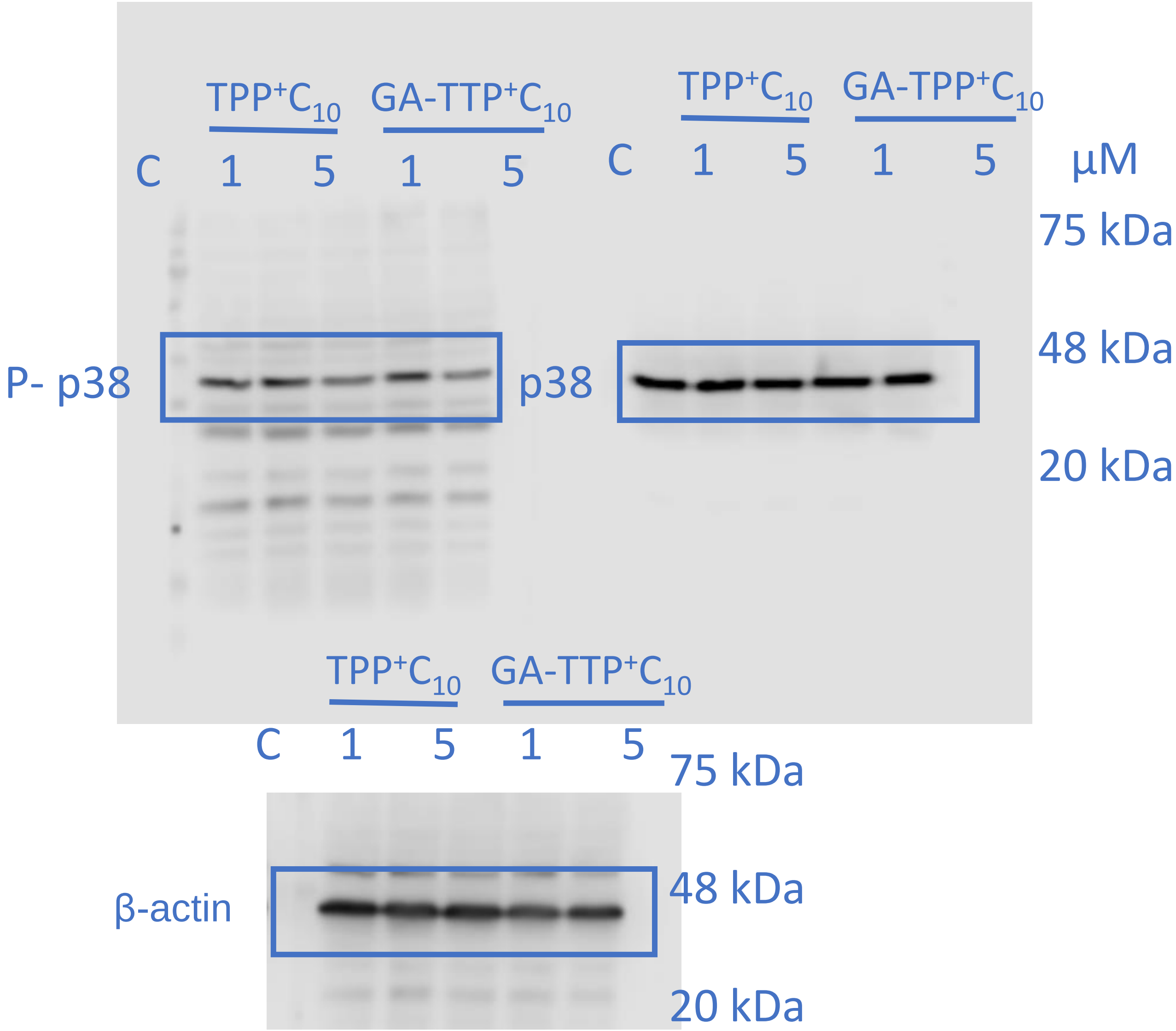

TPP⁺C10 GA-TTP⁺C10
C 1 5 1 5
P- p38
TPP⁺C10 GA-TPP⁺C10
C 1 5 1 5 µM
75 kDa
48 kDa
p38
20 kDa
TPP⁺C10 GA-TTP⁺C10
C 1 5 1 5
75 kDa
48 kDa
β-actin
20 kDa

Figure 5 E

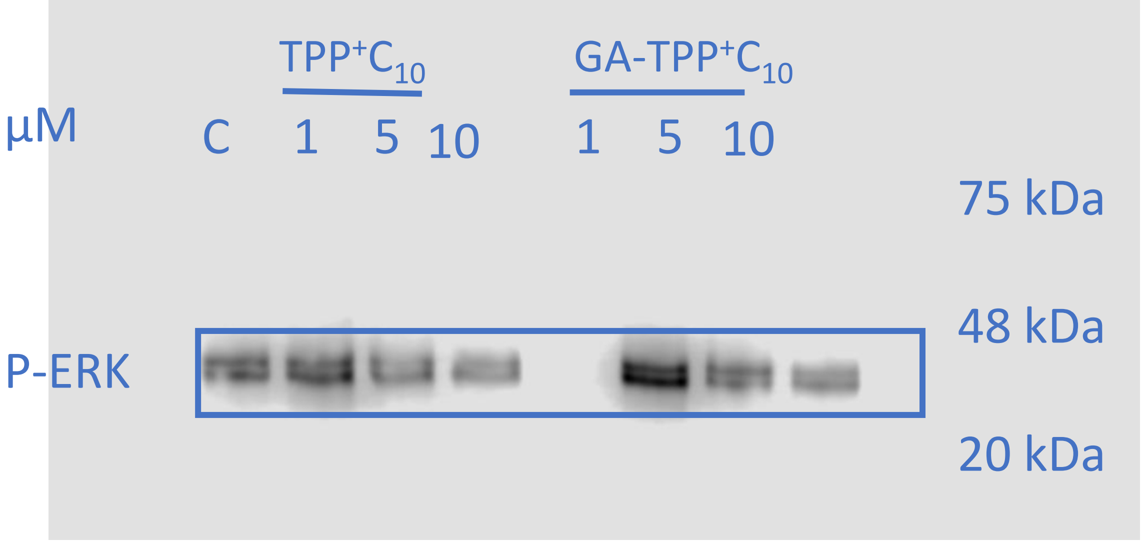

TPP+C10
GA-TPP+C10
µM
C 1 5 10 1 5 10
75 kDa
48 kDa
P-ERK
20 kDa

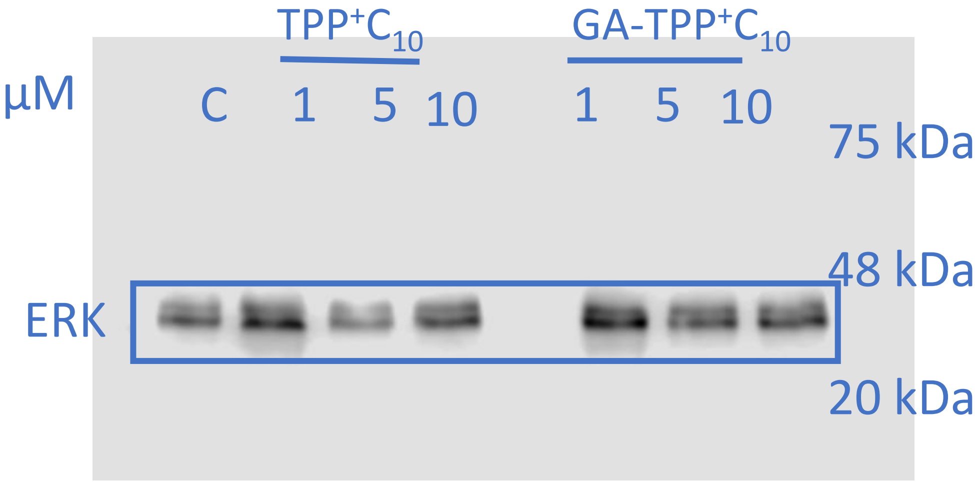

TPP+C10
GA-TPP+C10
µM
C 1 5 10 1 5 10
75 kDa
48 kDa
ERK
20 kDa

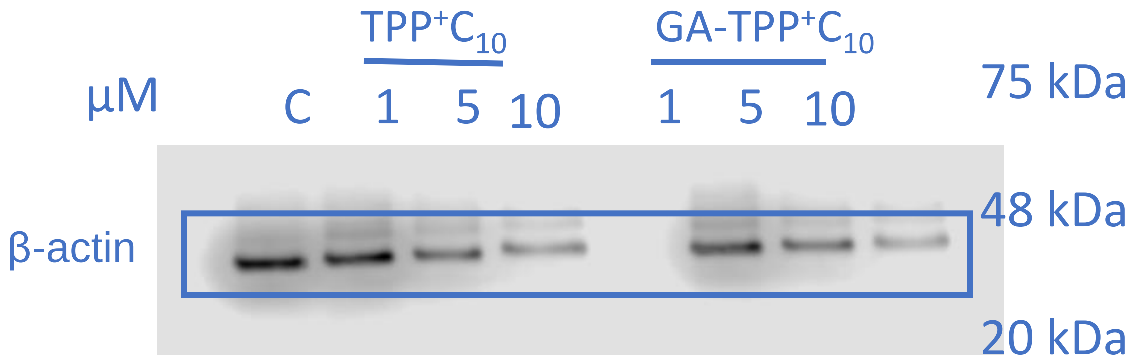

TPP+C10
GA-TPP+C10
µM
C 1 5 10 1 5 10
75 kDa
48 kDa
β-actin
20 kDa

Figure 5 E

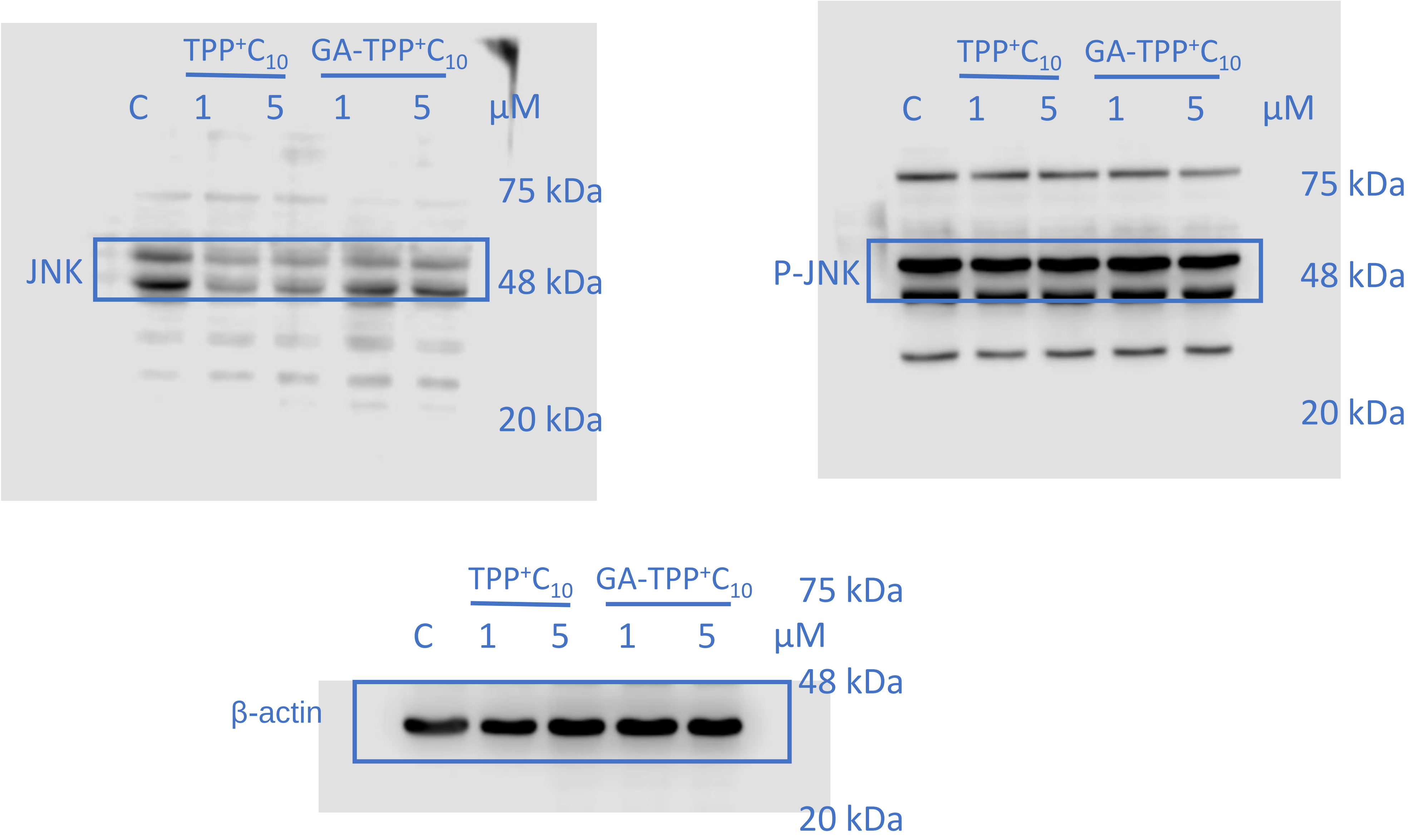

TPP+C10
GA-TPP+C10
C 1 5 1 5 µM
75 kDa
JNK
48 kDa
20 kDa
TPP+C10
GA-TPP+C10
C 1 5 1 5 µM
75 kDa
P-JNK
48 kDa
20 kDa
TPP+C10
GA-TPP+C10
75 kDa
C 1 5 1 5 µM
48 kDa
β-actin
20 kDa

Figure 5 E

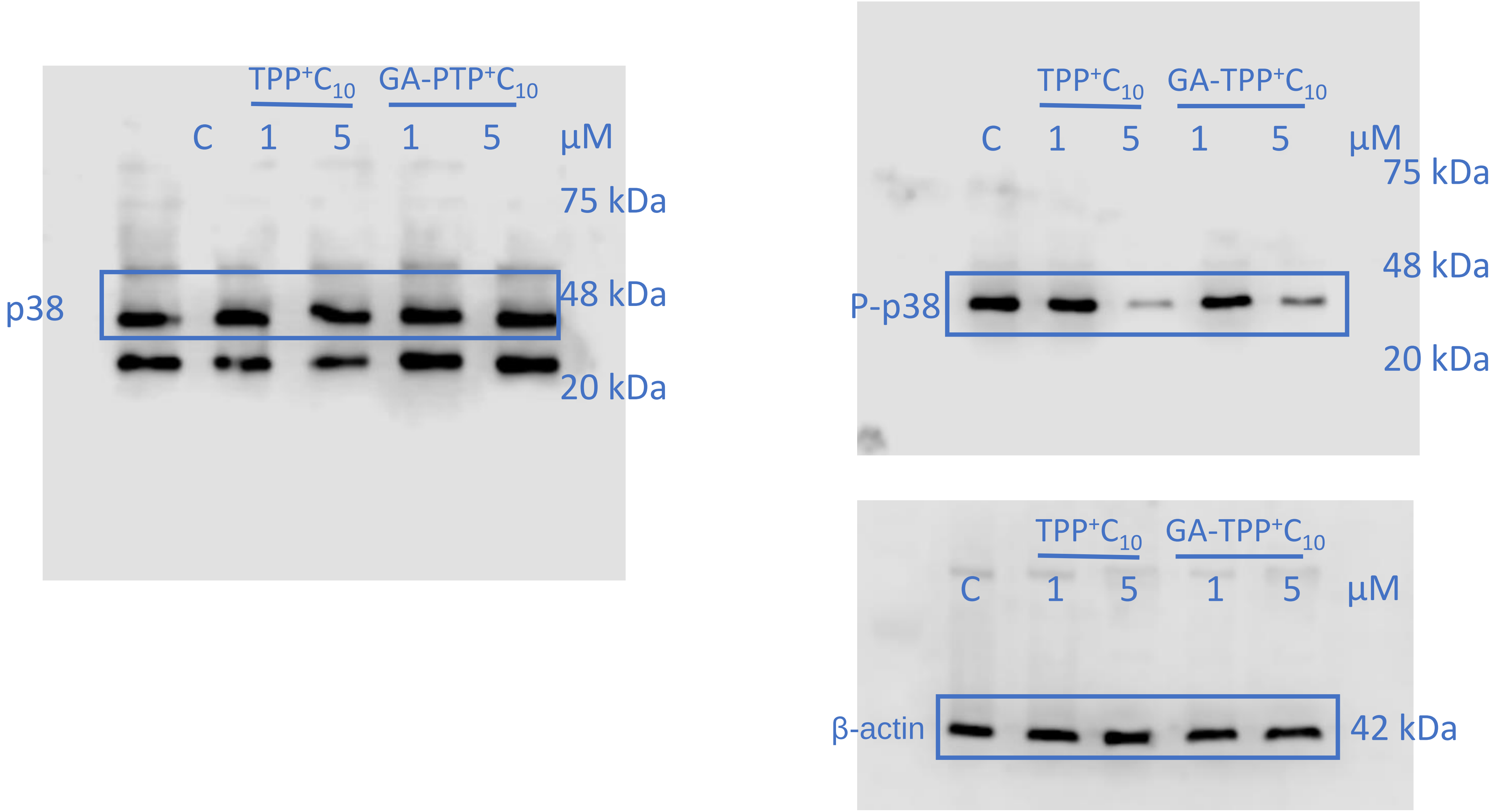

TPP+C10
GA-PTP+C10
C 1 5 1 5 µM
75 kDa
48 kDa
20 kDa
p38
TPP+C10
GA-TPP+C10
C 1 5 1 5 µM
75 kDa
48 kDa
20 kDa
P-p38
TPP+C10
GA-TPP+C10
C 1 5 1 5 µM
β-actin
42 kDa

Figure 6 A

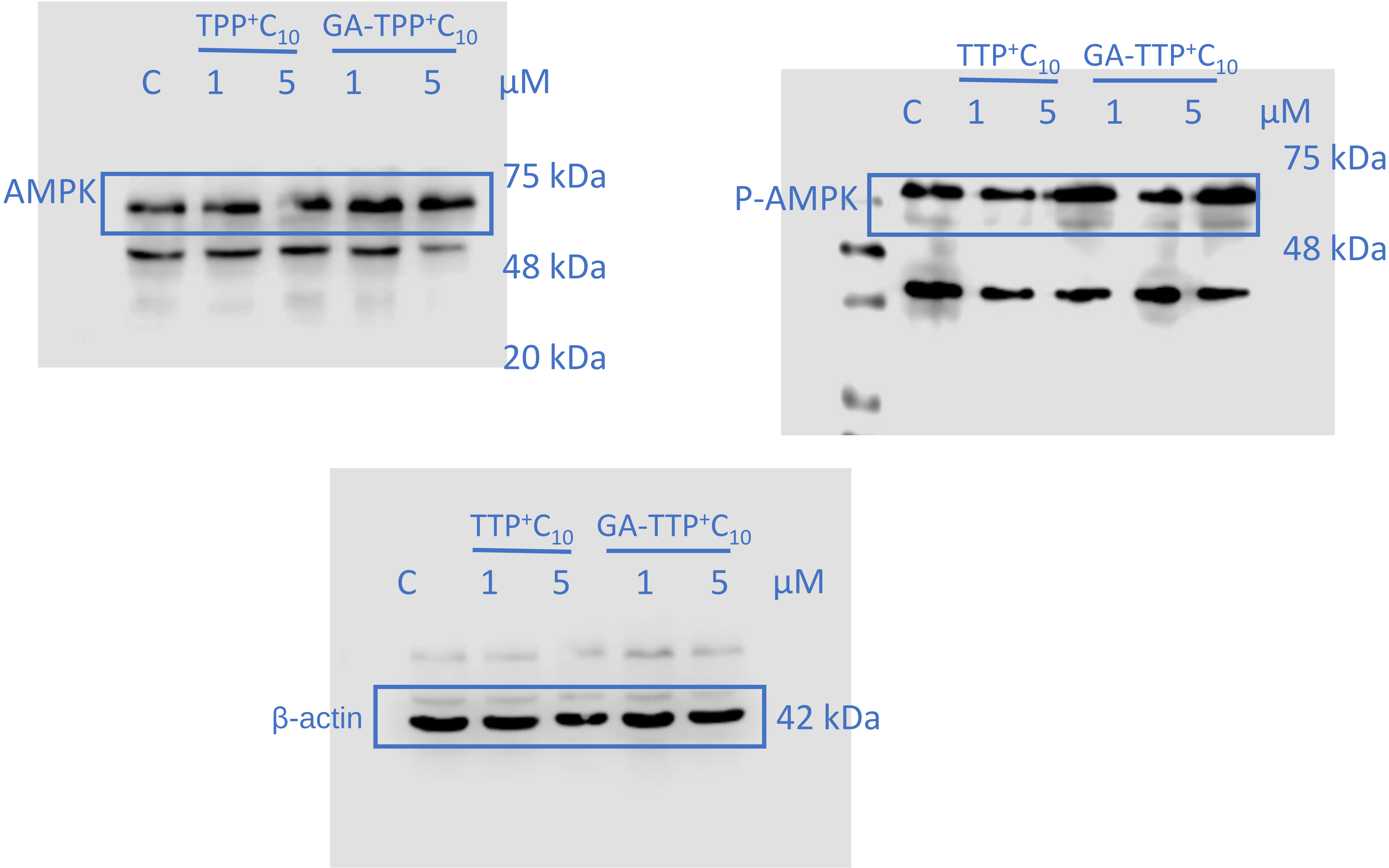

TPP+C10
GA-TPP+C10
C 1 5 1 5 µM
AMPK
75 kDa
48 kDa
20 kDa
TTP+C10
GA-TTP+C10
C 1 5 1 5 µM
P-AMPK
75 kDa
48 kDa
TTP+C10
GA-TTP+C10
C 1 5 1 5 µM
β-actin
42 kDa

Figure 6 A

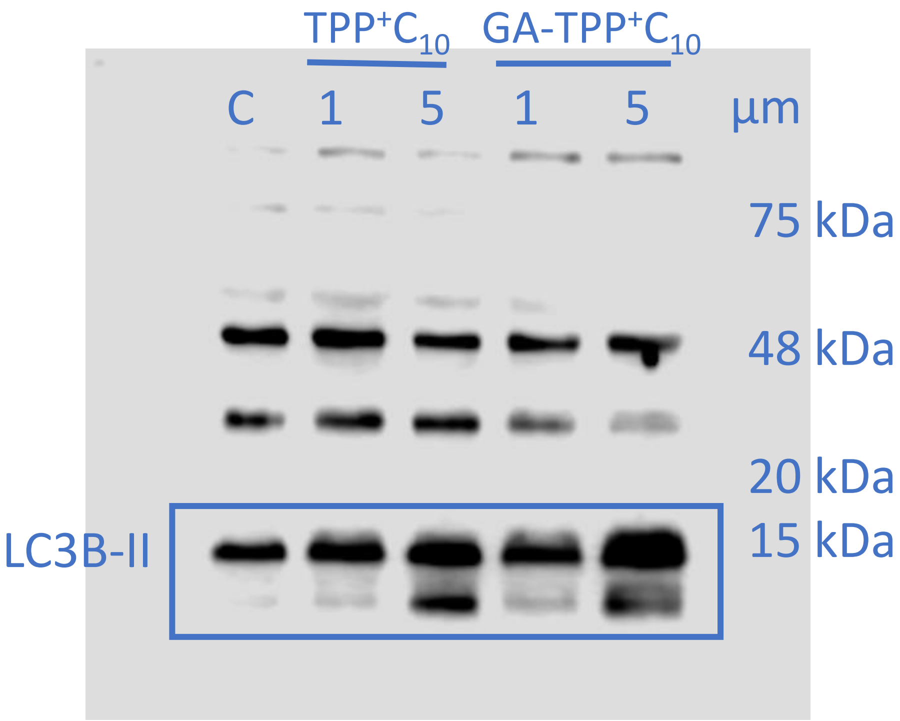

TPP+C10
GA-TPP+C10
C 1 5 1 5 µm
75 kDa
48 kDa
20 kDa
15 kDa
LC3B-II

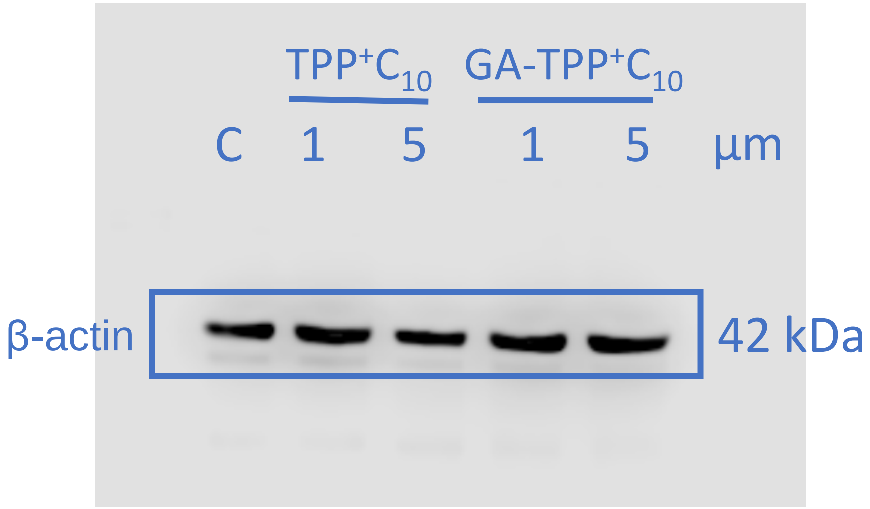

TPP+C10
GA-TPP+C10
C 1 5 1 5 µm
β-actin
42 kDa

Figure 6 D

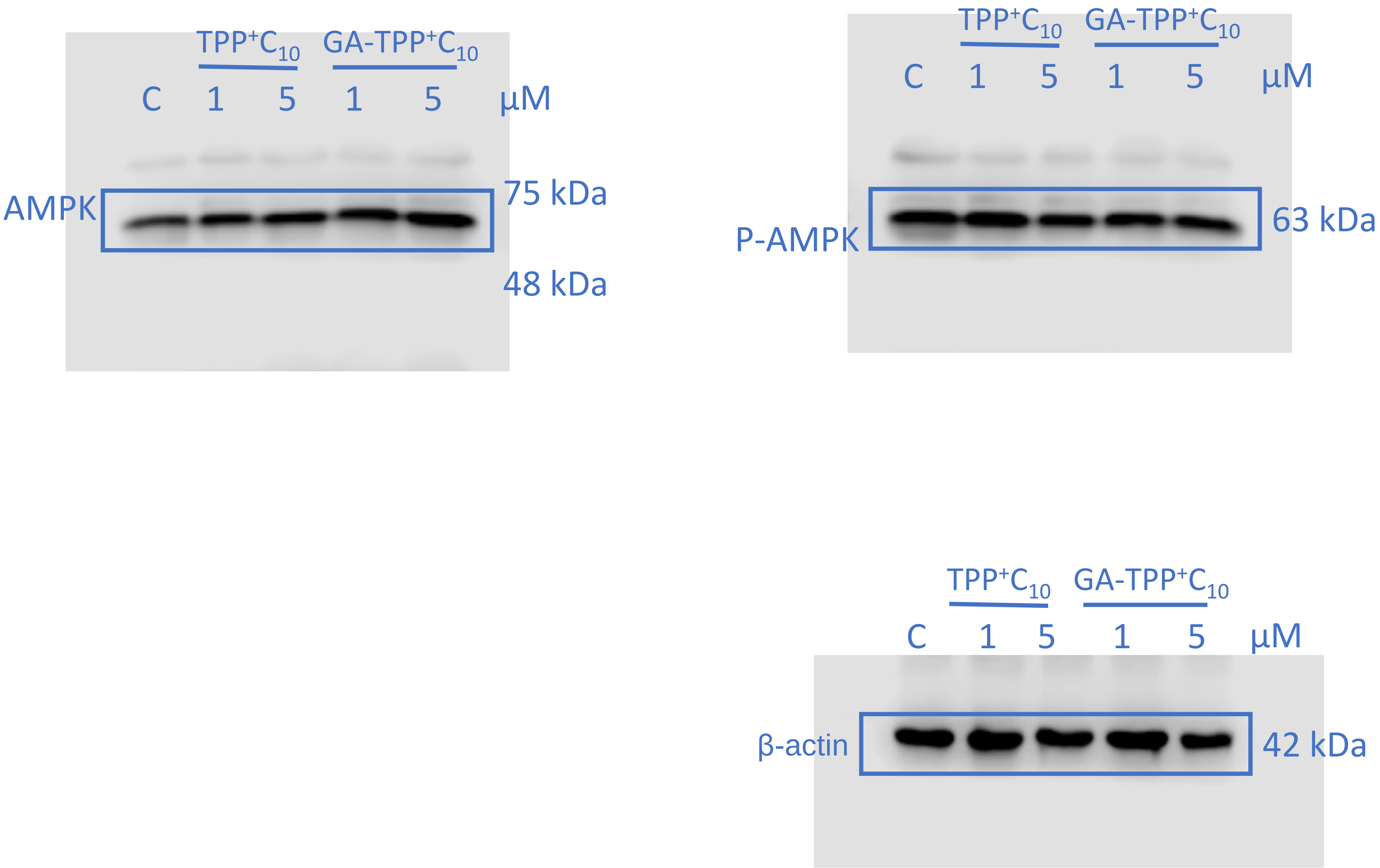
TPP+C10
GA-TPP+C10
C 1 5 1 5 µM
AMPK
75 kDa
48 kDa
P-AMPK
63 kDa
β-actin
42 kDa

Figure 6 D

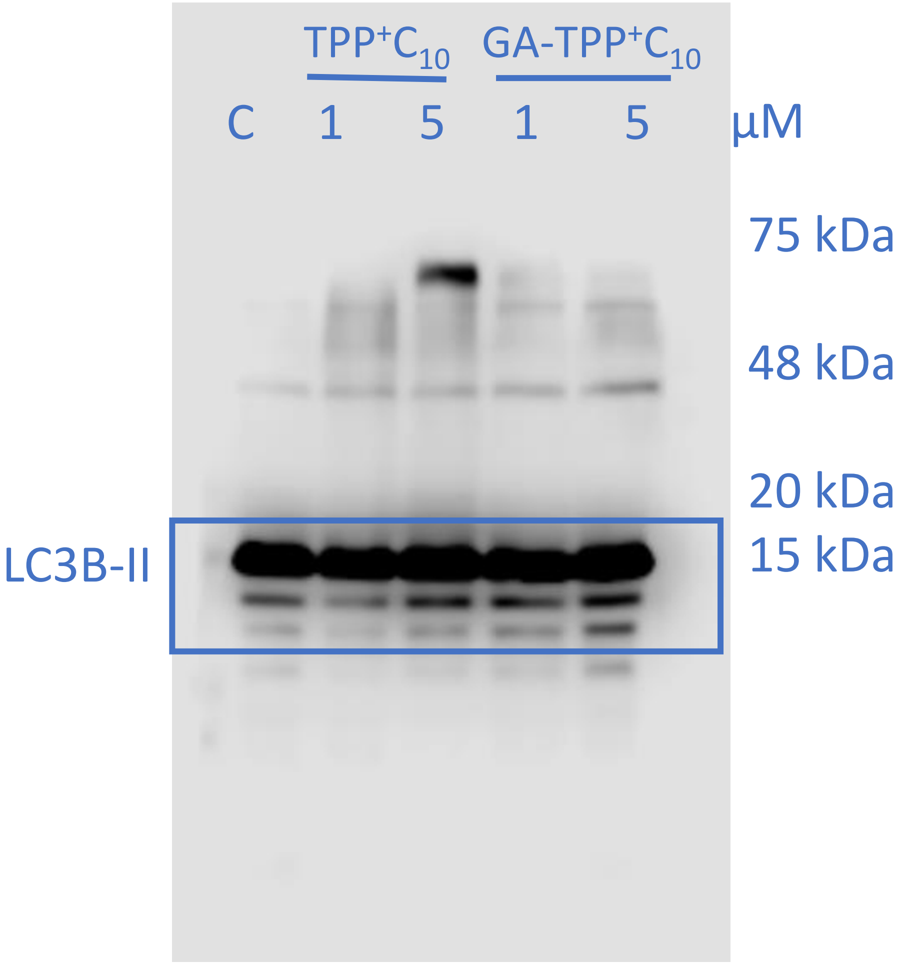

TPP+C10
GA-TPP+C10
C 1 5 1 5 µM
75 kDa
48 kDa
20 kDa
15 kDa
LC3B-II

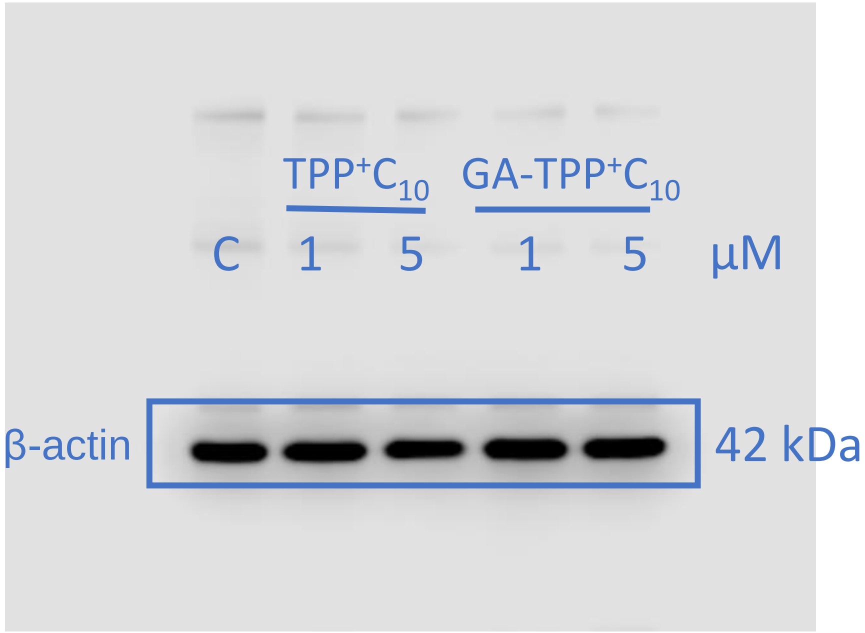

TPP+C10
GA-TPP+C10
C 1 5 1 5 µM
β-actin
42 kDa

Figure 6 G

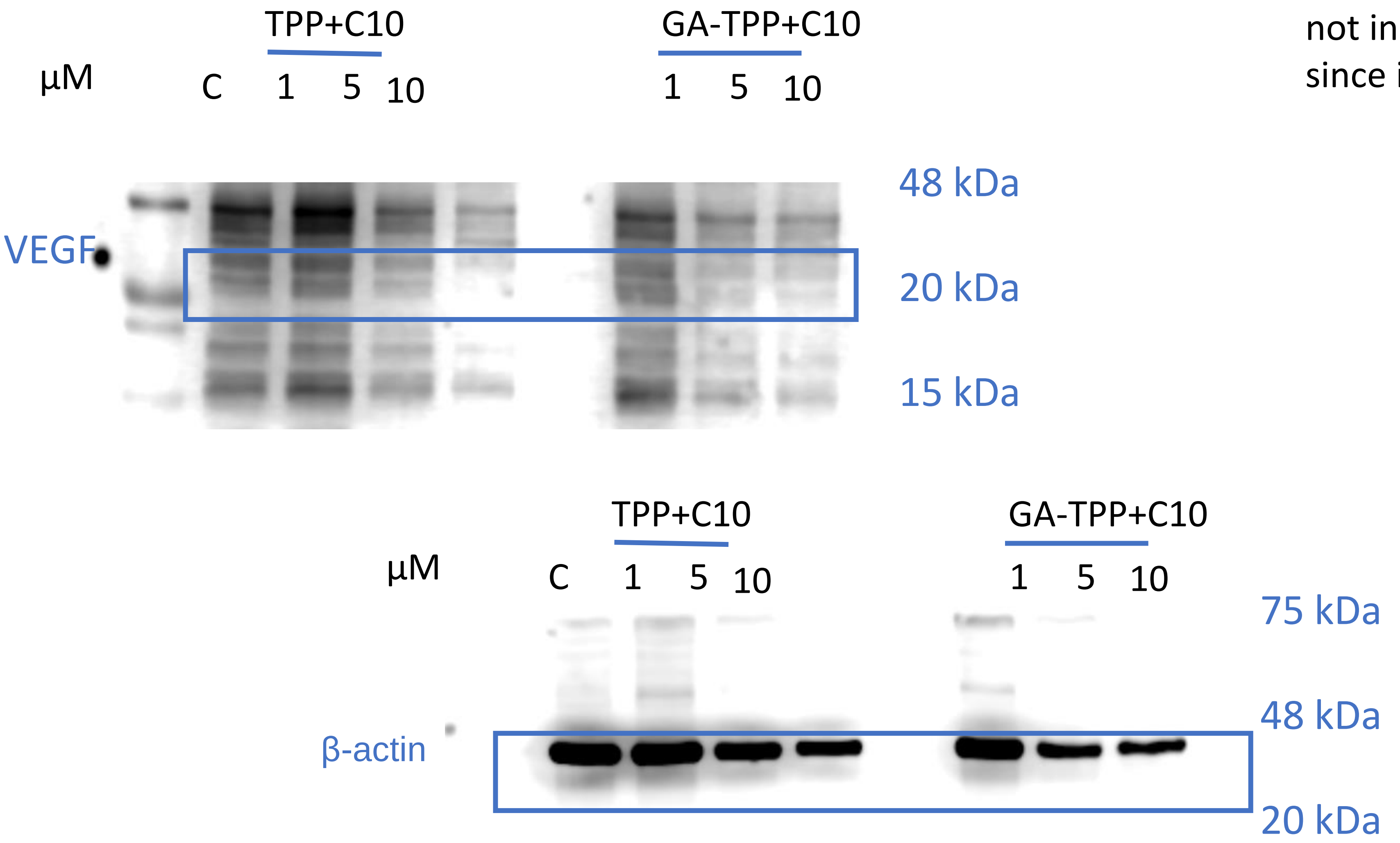

The concentration of 10 uM was not included in the final blot, since it reduce cell viability

Figure 6 J

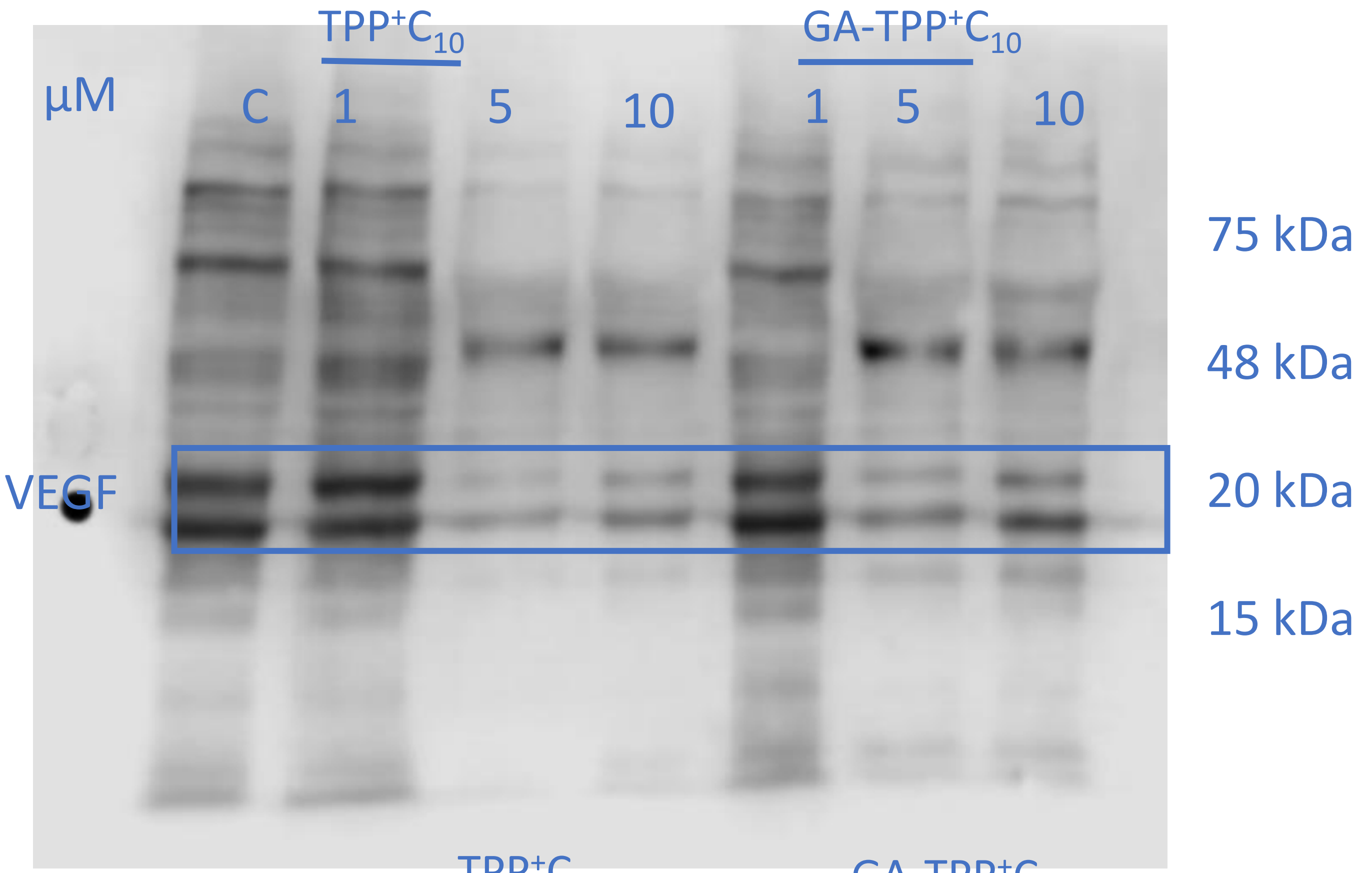

The concentration of 10 uM was not included in the final blot, since it reduce cell viability

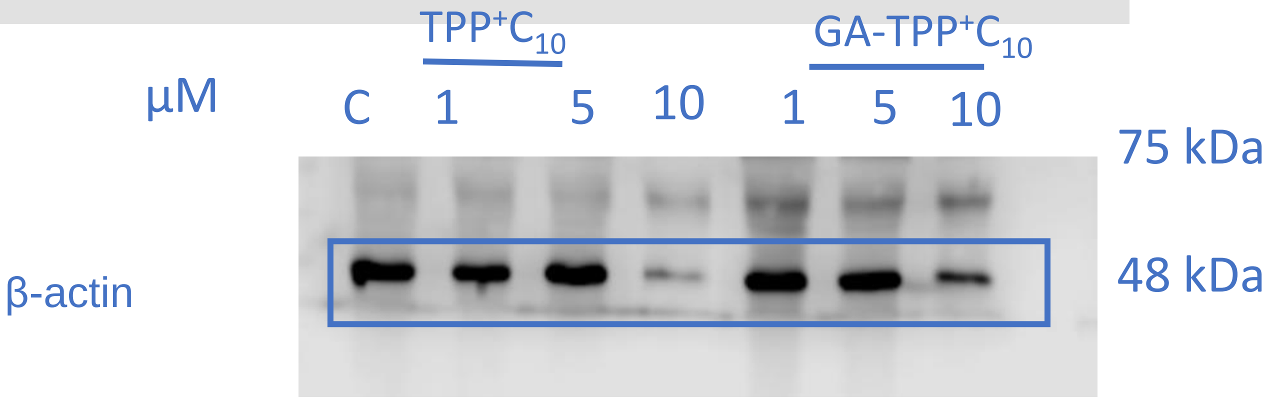

Supplement: Supplementary file 1 [file cancers-16-02980-s001.zip › cancers-3148539-supplementary/cancers-3148539-supplementary/cancers-3148539-Western Blot.pdf]
